# Bipartite Genetically Encoded Biosensors to Sense Calcium Ion Dynamics at Membrane–Membrane Contact Sites

**DOI:** 10.1021/acs.analchem.5c03831

**Published:** 2025-09-01

**Authors:** Issei Yamaguchi, Lucia Barazzuol, Giulia Dematteis, Wenchao Zhu, Yurong Wen, Mikhail Drobizhev, Dmitry Lim, Robert E. Campbell, Tito Calì, Yusuke Nasu

**Affiliations:** 1 Department of Chemistry, School of Science, 13143 The University of Tokyo, Bunkyo-ku, Tokyo 113-0033, Japan; 2 Department of Biomedical Sciences, University of Padova, Padova 35131, Italy; 3 Department of Pharmaceutical Sciences, Università del Piemonte Orientale, Novara 28100, Italy; 4 Center for Microbiome Research of Med-X Institute, The First Affiliated Hospital, Xi’an Jiaotong University, Xi’an, Shaanxi 710061, China; 5 Department of Microbiology and Cell Biology, 33052 Montana State University, Bozeman, Montana 59717, United States; 6 CERVO Brain Research Centre, Québec, Quebec G1J 2G3, Canada; 7 Department of Biochemistry, Microbiology, and Bio-informatics, Laval University, Québec, Quebec G1 V 0A6, Canada; 8 Padova Neuroscience Center, University of Padova, Padova 35131, Italy; 9 Study Center for Neurodegeneration, University of Padova, Padova 35131, Italy; 10 Institute of Biological Chemistry, 38017 Academia Sinica, Nankang, Taipei 115, Taiwan; 11 Institute of Biochemical Sciences, National Taiwan University, Da’an, Taipei 106, Taiwan

## Abstract

Self-complementing bipartite fluorescent proteins (FPs) are useful tools for the detection of protein–protein proximity and for localizing fluorophores to membrane–membrane contact sites. Here, we report versions of circularly permuted green FP (GFP), red FP (RFP), and mNeonGreen (NG), which are split into a large fragment composed of nine β-strands and a small fragment composed of two β-strands. In each case, the large and small fragments can associate in live cells to form the complete 11-stranded FP β-barrel. We further converted each of these three self-complementing FPs into bipartite calcium ion (Ca^2+^) biosensors. We demonstrate that appropriately targeted versions of these split FPs, and split FP-based biosensors, can be functionally assembled at membrane–membrane contact sites. We employ the bipartite NG-based Ca^2+^ biosensor for visualization of pharmacologically induced Ca^2+^ release at mitochondria-endoplasmic reticulum contact sites (MERCs).

## Graphical abstract

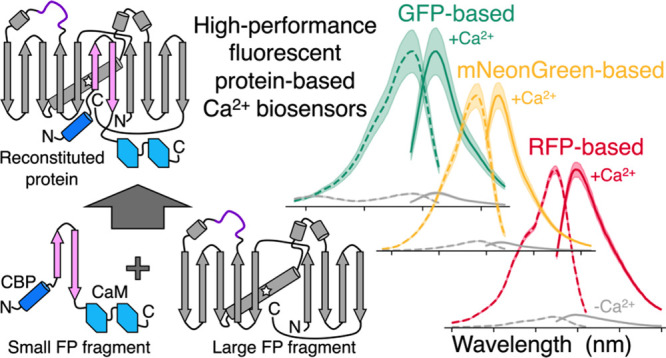

## Introduction

Self-complementing bipartite FPs are versatile tools for a range of cellular imaging applications including labeling of endogenous proteins,^1^ labeling of cell–cell contact sites,^2^ and directing of the self-assembly of protein nanostructures,^3^ among others.^4,5^ We use the term *self-complementing* to refer to the ability of two separate polypeptide chains (i.e., fragments) to spontaneously associate and form an intact protein (i.e., a split or bipartite protein). To date, a wide variety of designs of bipartite FPs has been developed and used in various applications. The first example of split *Aequorea* GFP was reported in 2000 by Ghosh et al.^6^ and was not self-complementing. In this system, GFP (composed of 11 β-strands) was split between the seventh and eighth β-strands (between residues 157 and 158), and complementation was dependent on the interaction of a fused heterodimerizing pair of proteins. An example of a self-complementing GFP was reported in 2005 by Cabantous et al.^7^ In this system, GFP was split between the 10th and 11th β-strands (between residues 214 and 215) (Figure 1a). In later work, this design was extended to other FPs including color variants of GFP,^8,9^ mNeonGreen,^10,11^ superfolder mCherry,^1,9,12^ mScarlet,^13^ and mRuby.^9^ The large majority of these designs have employed the FP in its normal topology, with a large fragment composed of β-strands 1–10 (FP1-10) and a small fragment composed of β-strand 11 (FP11). Systems that require both FP10 and FP11, either separately^14^ or as a FP10-11 fusion,^15^ have also been reported.

**Figure 1:**
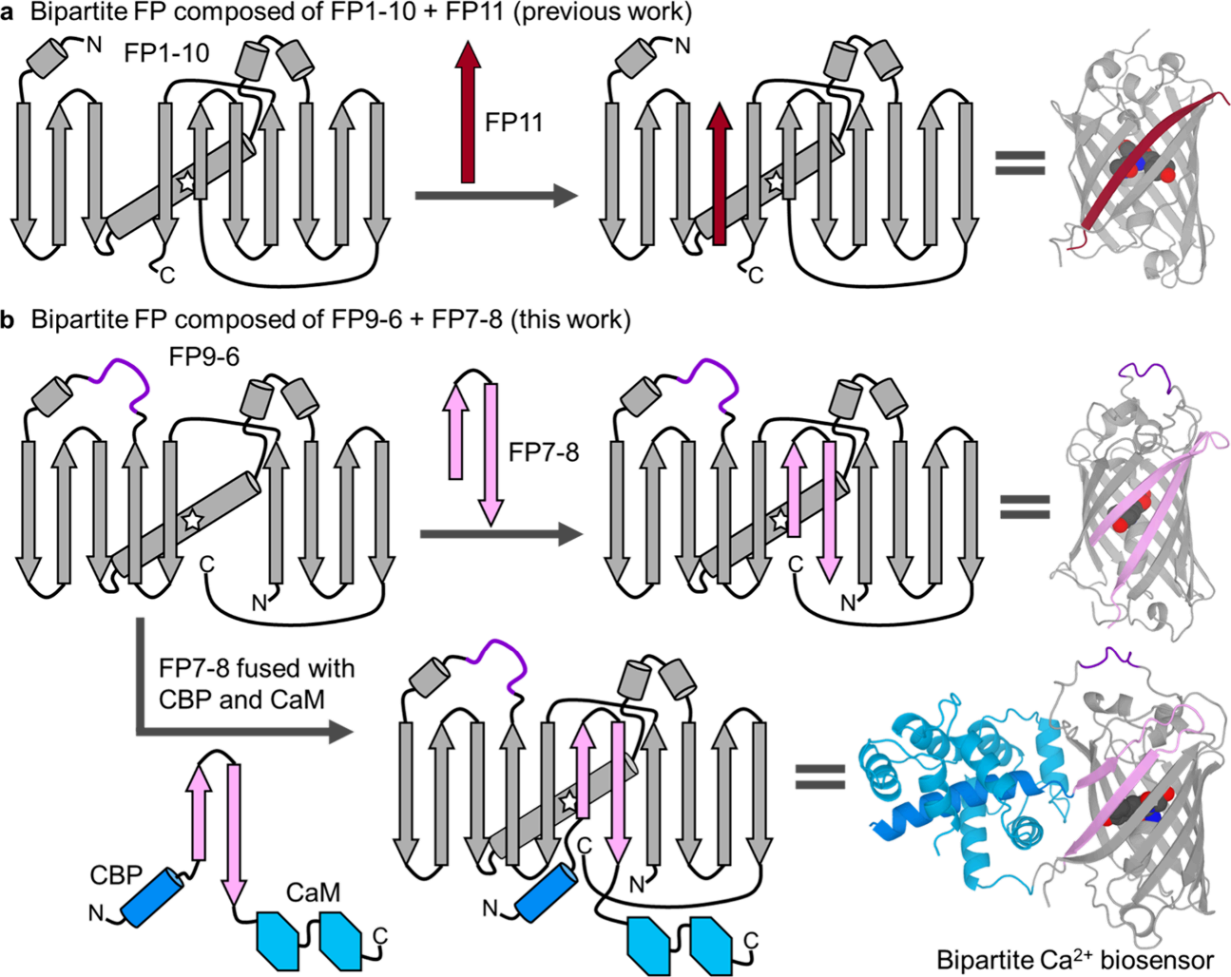
Strategies for the development of bipartite FPs and FP-based biosensors. The overall fold of an archetypical FP is represented using arrows for β-strands, cylinders for α-helices, and a star to represent the chromophore.^32^ (a) In previous work, bipartite FPs have typically been developed by splitting them into a large fragment containing β-strands 1 through 10 (FP1-10; gray) and a small fragment containing β-strand 11 (FP11; dark red). (b) In this work, we created bipartite cpFPs with a large fragment composed of nine β-strands (FP9-6; gray) and a small fragment composed of two β-strands (FP7-8; pink). To create bipartite Ca^2+^ biosensors, we fused FP7-8 to calmodulin (CaM; light blue) and a CaM-binding peptide (CBP; dark blue).

There have been relatively few efforts to create bipartite FPs in which internal β-strands (that is, strands other than FP11) have been used as the small fragment.^9,16^ In order to use an internal β-strand as the small fragment, the topology of the FP must first be modified to a circularly permuted (cp) version.^17^ In cpFP, the gene has been rearranged such that the original N- and C-termini are linked (via a cp linker) and new termini are introduced elsewhere in the protein. Tamura et al.^9^ found that a fluorescent bipartite cpGFP could be produced using the combinations FP8-6 + FP7, FP9-7 + FP8, and FP11-9 + FP10. In this notation, FP8-6 represents a topological variant of an FP with β-strands on the order of N-8-9-10-11-cplinker-1-2-3-4-5-6-C, for example.

Although there are many examples of different bipartite FPs, with different colors and different split sites, there is a notable lack of bipartite self-complementing single FP-based biosensors. Single FP-based biosensors are constructed by genetic insertion of a responsive domain (e.g., a domain that undergoes a Ca^2+^-dependent conformational change) in a privileged location (i.e., the “bulge region”)^18^ in the seventh β-strand of the FP. A conformational change in the responsive domain changes the FP chromophore environment, leading to changes in the fluorescence intensity. The archetypical examples of such biosensors are the GCaMP series of GFP-based Ca^2+^ biosensors,^19–21^ and the color palette of Genetically Encoded Ca^2+^ biosensor for Optical imaging (GECO).^22–24^ Self-complementing FPs have been incorporated into Förster resonance energy transfer (FRET)-based biosensors.^25^ Notable examples of bipartite biosensors include the Sphere-SF-iGluSnFR glutamate biosensor^26^ and the split version of the FRCaMP and GCaMP Ca^2+^ biosensor.^27,28^ These biosensors were split between the sixth and seventh β-strands, and in all cases, the two fragments had to be fused to a heterodimerizing pair of proteins to make them self-complementing.

A shortcoming of previously reported split biosensors is that they are not inherently self-complementing. We reasoned that a split FP, in which the small fragment was composed of the seventh and eighth β-strands, would be particularly amenable to conversion into a self-complementing biosensor (Figure 1b). Importantly, the N- and C-termini of this small fragment would be close together in space and close to the critical bulge region. Accordingly, we expected that a conformational change of a responsive domain fused to the termini of this small fragment could induce changes in the fluorescence intensity.

In an effort to realize our design, and create a new strategy for construction of self-complementing bipartite FPs and FP-based biosensors, we developed versions of EGFP,^29^ mApple,^30^ and mNeonGreen (NG)^31^ that are split into a nine-β-strand fragment (FP9-6; N-9-10-11-cplinker-1-2-3-4-5-6-C) and a two β-strand fragment (FP7-8) (Figure 1b). Starting from each of these split FPs, we have created split Ca^2+^ biosensors designated split green (sG)-GECO1, split red (sR)-GECO1, and split neon (sN)-GECO1, respectively.

## Experimental Section

### Engineering of Self-Complementing Bipartite scpFPs

To develop self-complementing bipartite scpGFP1 and scpRFP1, we first synthesized the DNAs encoding cpGFP and cpmApple of iGluSnFR^33^ and R-GECO1 (ref^22^), respectively, and cloned them between the *XhoI* and *HindII* sites of pBAD/His B. To mimic a bipartite protein using a single polypeptide chain, these cpGFP and cpmApple genes contain a long “spacer” sequence (32 a.a.: DVGGGGSEGGGSGGPGSGGEGSAGGGSAGGGS)^10^ between the eighth and ninth strands. Using GFP numbering, the cpGFP construct began with an appended methionine immediately before residue 147 and ended with residue 146 (Figure S1). Using RFP numbering, the cpRFP construct began with a methionine before residue 146 and ended with residue 145 (Figure S3). To create genetic libraries, the whole gene encoding cpGFP and cpmApple including spacer were randomized by error-prone PCR to satisfy an error rate of one to two amino acid substitutions in each round. From each round, the top three to five brightest colonies on the LB plate from more than 1000 colonies were selected as the templates for the next round. Three and eight rounds of iterative library creation and screening led to the finalization of scpGFP1 and scpRFP1, respectively.

To develop self-complementing bipartite scpNG1, the gene encoding mNG-GECO1 (ref 24) was used as the template. We first constructed cpNG using PCR amplified NG7-11 and NG1-6 of mNG-GECO1, and the reported polypeptide sequence (VDGGSGGTG)^34^ as a cp linker. NG7-8 and NG9-6 were transplanted under the first and second RBS of the pBiC plasmid, respectively. pBiC is a bicistronic plasmid that was previously named as bic-pBAD and was a kind gift from Jhon R. Enterina.^35^ The resulting prototype was designated as scpNG0.1. The whole gene encoding scpNG0.1 including second RBS was randomized by error-prone PCR. As described above, we performed four rounds of directed evolution and finally obtained scpNG1.

To construct the prototype of the scpGFP1-based bipartite Ca^2+^ biosensor, we first fused CBP and CaM, which both derived from jGCaMP7s,^20^ to the N- and C-termini of the GFP7-8 of scpGFP1 via the reported^20^ “LE” and a flexible “GGGG” linker, respectively. The fragment containing CBP, GFP7-8, and CaM is located under the first RBS, and the other fragment containing GFP9-6 is located under the second RBS in the pBiC plasmid. We subsequently optimized the linkers by combining linker deletions for both linkers and conducting site-saturation mutagenesis of both linkers and their surrounding residues, which identified the variant with no linker at the first linker and “SLR” at the second linker performed the best at that time. We then performed six rounds of iterative directed evolution on this variant and finally established sG-GECO1 (Figures S1 and S7).

To construct the prototype of the scpNG1-based bipartite Ca^2+^ biosensor, we first fused CBP and CaM, which both derived from sG-GECO1, to the N- and C-termini of NG7-8 via flexible “GG” linkers at both termini, as we did in development of sG-GECO1 above. To enhance the Δ*F*/*F*_min_ and biosensor brightness, we first optimized the linker sequences by site-saturation mutagenesis. Sequential screening of the linker-randomized library found the variant with “GLMDW” at the first linker and “ES” at the second linker showed the best performance. Then, we performed one round of directed evolution on the whole gene region of this variant and found beneficial mutations (Phe45Ser/Ala211Val). We noticed the linker length between CBP and NG7-8 (defined as amino acid between the end of CBP and the “gate post” residue Asn29) is relatively longer than sG-GECO1, or mNG-GECO1. Thus, we tested five different linker lengths and found that the deletion of Met held the best fluorescence properties. We then performed four rounds of iterative directed evolution on the whole gene, followed by the final gene shuffling by the Staggered extension process^36^ on four found candidates. These processes ultimately established sN-GECO1 (Figures S2 and S7).

To construct the prototype of the scpRFP1-based bipartite Ca^2+^ biosensor, we first fused CBP and CaM, which both derived from R-GECO1 (ref^22^), to the N- and C-termini of RFP7-8 of scpRFP1 via the reported “PV” and a flexible “GGG” plus reported “TR” linkers, respectively. To enhance Δ*F*/*F*_min_ and biosensor brightness, we then optimized the length of each linker and found that the deletion of “P” and “GG” worked the best at that time. We next performed site-saturation mutagenesis at both linkers separately. Screening of these libraries found that the variant with “S” at the first linker and “VER” at the second linker showed the best performance. We then performed eight rounds of iterative directed evolution on this variant and finally established sR-GECO1 (Figures S3 and S7).

### Purification and *In Vitro* Characterization of scpFPs

To perform *in vitro* characterization, we transferred small and large fragments of scpGFP1 and scpRFP1 to pBiC plasmids by enzymatic digestion and ligation. The genes encoding the protein of interest with a 6×-histidine tag on the N-terminus of the first fragment were expressed from pBiC. Transformed bacteria were cultured in Terrific Broth supplemented with 100 μg mL^–1^ of ampicillin. After an optical density reached around 0.8, the culture was supplemented with l-arabinose to the final concentration of 0.04% and continued to be cultured 1 or 2 days at room temperature or 17 °C. Bacteria were collected by centrifugation and lysed with a cell disruptor (Branson). Extracted proteins were purified by Ni-NTA affinity chromatography (G-Biosciences) according to the manufacturer’s recommended protocol. The eluted proteins were concentrated and dialyzed into an exchange buffer (100 mM KCl, 10 mM MOPS at pH7.2) with an Amicon Ultra-0.5 Centrifugal Filter Device (Millipore).

Absorbance, excitation, and emission spectra and quantum yield (QY) were recorded with diluted protein samples. Molar extinction coefficients (ECs) were calculated as described previously.^37^ pH titration was carried out by 5 μL of diluting concentrated protein samples with 50 μL of buffers with pH ranging from 3.0 to 11.5 containing 30 mM trisodium citrate, 30 mM sodium borate, 30 mM MOPS, and 100 mM KCl. Fluorescence intensities as a function of pH were fitted to a monophasic dose–response curve to determine the apparent p*K*_a_.

To quantitatively evaluate the brightness of the evolved variants on the LB plate (as in Figure 2b,f,j), we streaked the bacteria expressing each variant and captured the images using a custom filter and camera set (colony screener). The images were analyzed by using a custom Python script to calculate the relative brightness of each construct on the LB plate.

**Figure 2:**
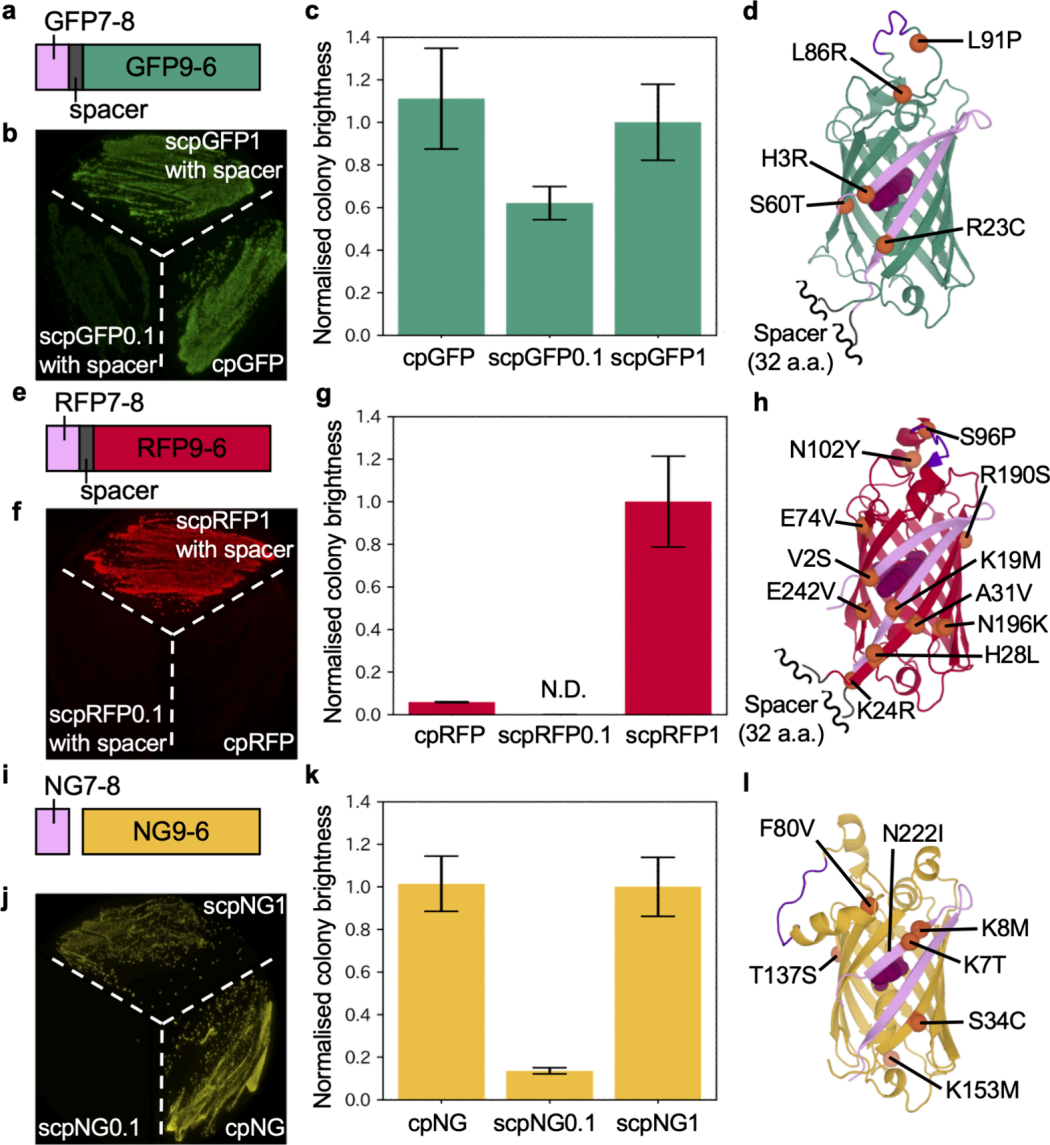
Development of self-complementing bipartite scpFPs. (a–d) Development of scpGFP1. (a) In scpGFP0.1, a 32-amino-acid spacer was used to connect GFP7-8 and GFP9-6. (b) Fluorescence image of an LB plate with streaked bacteria expressing scpGFP0.1 (with a spacer), scpGFP1 (with a spacer), and cpGFP. (c) When expressed as separate polypeptides (GFP7-8 and GFP9-6) from a single plasmid in bacterial colonies, scpGFP1 exhibited 90% of the brightness of cpGFP itself.^39^ Values were normalized to scpGFP1 and are given as mean ± standard deviation (sd) (*n* > 1000). (d) Structural prediction of scpGFP1, with the spacer insert. The positions of mutations acquired during directed evolution are shown as orange spheres and labeled. The GFP7-8 fragment, the cp linker, and the chromophore are colored in pink, purple, and dark purple, respectively. (e–h) Development of scpRFP1. (e) scpRFP0.1, as in (a). (f) Fluorescence images of bacteria expressing scpRFP0.1 (with spacer), scpRFP1 (with spacer), and cpRFP. (g) In bacterial colonies, scpRFP1 exhibited 1700% of the brightness of cpRFP. Data was processed as described in (c) (*n* > 700). (h) Structural prediction of scpRFP1, as in (d). (i–l) Development of scpNG1. (i) The initial prototype of scpNG (scpNG0.1) was expressed as separate polypeptides. (j) Fluorescence image of bacteria expressing scpNG0.1, scpNG1, and cpNG. (k) When expressed as separate polypeptides in bacterial colonies, scpNG1 exhibited 99% of the brightness of cpNG. Data was processed as described in (c) (*n* > 100). (l) Structural prediction of scpNG1, as in (d).

To confirm that each of the scpFPs is orthogonal to each other (as in Figure 3b), we transferred all combinations of a small fragment and a large fragment of scpGFP1, scpNG1, and scpRFP1, into pBiC plasmids using enzymatic digestion and ligation. Bacteria expressing each combination were streaked on LB plates, and images were captured.

**Figure 3:**
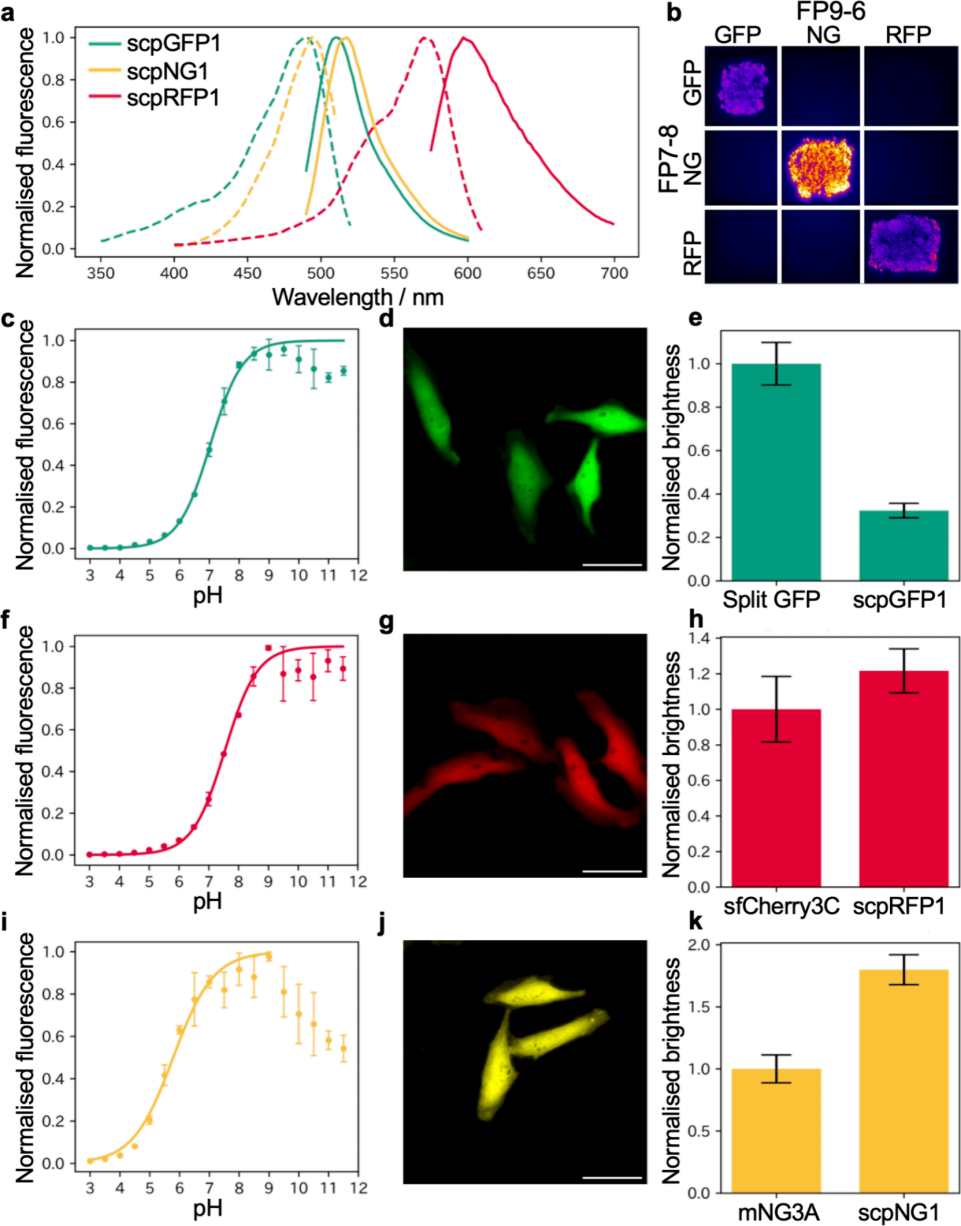
Characterization of scpFPs. (a) Excitation (dashed line) and emission spectra (solid line) of purified scpGFP1 (green), scpNG1 (yellow), and scpRFP1 (red). (b) Fluorescence image of an LB plate streaked with bacteria expressing all combinations of FP7-8 and FP9-6 from scpGFP1, scpNG1, and scpRFP1. The polypeptides were expressed using the pBiC plasmid with two RBSs. (c–e) Characterization of scpGFP1. (c) pH titration curve. *n* = 3 independent experiments (mean ± sd). (d) Fluorescence image of HeLa cells expressing scpGFP1, when expressed as two polypeptides with an intervening P2A self-cleaving tag. Scale bar is 50 μm. (e) Relative brightness of scpGFP1 versus a previously reported FP1–10 + FP11 system (“Split GFP”),^7^ when expressed in HeLa cells as described in (d). Mean ± standard error (sem) (*n* = 138 and 139 cells, respectively). (f–h) Characterization of scpRFP1, as for scpGFP1 in (c–e). In (h), the brightness of scpRFP1 is compared to the previously reported sfCherry3C system^12^ composed of FP1-10 + FP11 fragments (*n* = 167 and 78 cells, respectively). (i–k) Characterization of scpNG1, as for scpGFP1 in (c–e). In (k), the brightness of scpNG1 is compared to the previously reported mNG3A system^11^ composed of FP1-10 + FP11 fragments (*n* = 156 and 122 cells, respectively).

### Purification and *In Vitro* Characterization of sGECO Ca^2+^ Biosensors

sG-, sN-, and sR-GECO1 expressed from pBiC plasmids were purified as described above. Absorbance, excitation, and emission spectra and QY were recorded with concentrated protein samples mixed with buffers containing 100 mM KCl, 30 mM MOPS at pH 7.2, and 10 mM either EGTA or Ca-EGTA. ECs were calculated as described previously.^37^

To perform measurement of *K*_d,app_, we prepared a series of buffers containing 100 mM KCl, 30 mM MOPS at pH 7.2, and free [Ca^2+^] ranging from 0 to 39 μM, by mixing buffers used in the spectral measurements. Additionally, for the measurement of *K*_d,app_ of sR-GECO1, we also used high [Ca^2+^] buffers with free [Ca^2+^] ranging from 100 μM to 10 mM at pH 7.2, prepared by diluting a solution containing 100 mM KCl, 30 mM MOPS, and 10 mM CaCl_2_ into a solution containing 100 mM KCl and 30 mM MOPS. For sG- and sN-GECO1, Δ*F*/*F*_min_ were plotted to a monophasic dose–response curve (normalized Δ*F*/*F*_min_ = 1/(1 + (*K*_d,app_/[Ca^2+^])^hill^), where hill is a Hill coefficient), while Δ*F*/*F*_min_ of sR-GECO1 were plotted to a biphasic dose–response curve (normalized Δ*F*/*F*_min_ = frac/(1 + (*K*_d,app1_/[Ca^2+^])^hill1^ + (1 – frac)/(1 + (*K*_d,app2_/[Ca^2+^])^hill2^, where hill1 and hill2 are Hill coefficients for the first and second slope, respectively, and frac is the proportion of the maximal response of the two phases).

pH titration of sG-, sN-, and sR-GECO1 was performed by mixing 5 μL of concentrated protein samples with 50 μL of buffers with pH ranging from 3.0 to 11.5, containing 30 mM trisodium citrate, 30 mM sodium borate, 0.3 mM MOPS, and 1 mM KCl. For sG- and sN-GECO1, the buffers also included either 1 mM EGTA or 1 mM Ca^2+^, while sR-GECO1 used 10 mM Ca^2+^. Fluorescence intensities as a function of pH were fitted to a monophasic dose–response curve to determine the apparent p*K*_a_s.

### Ca^2+^ Measurements at ER-Mitochondrial Contact Sites

After the 8 h transfection, cells were washed three times with D-PBS, detached, and reseeded into μ-Slide 8 Well high (Ibidi; #80806). After 24 h, the cell medium was replaced by Krebs Ringer Buffer (KRB) supplemented with 0.1% d-glucose and 1 mM CaCl_2_ (Sigma-Aldrich; #21115), 150 μL per well. Using a Leica TSC SP5 inverted confocal microscope, single transfected cells were imaged for 100 s, one well at a time, one acquisition every 1.293 s. After the first 10 acquisitions, 50 μL of the same KRB solution was administered to each well. After another 15 acquisition, 50 μL of KRB solution with ATP and histamine (both 100 μM) was administered. The analysis was performed by using ImageJ (National Institutes of Health). Briefly, an ROI containing the cell of interest was drawn, and its mean intensity level was calculated for every single acquisition of the time-lapse.

### Cotransfection of SPLICa:ER-MT with ER-Mitochondrial Linkers

To investigate the effect of the ER-mitochondrial distance on the SPLICa:ER-MT-detected Ca^2+^ transients in MERCs, the SPLICa:ER-MT plasmid was cotransfected in HeLa cells with previously reported ER-mRFP (control), 10 nm ER-mitochondrial linker (EML), or 20 nm EML constructs.^38^ For this imaging, 4 × 10^4^ cells resuspended in 0.5 mL of complete medium were mixed with a transfection mix (0.5 mL of OptiMem (Gibco; #11058-021), 1.5 μg of each plasmid, and 1.5 μL of Lipofectamine 2000 (Invitrogen; #11668-019) and plated on 24 mm coverslips in six-well plates. Five hours after transfection, the medium was exchanged with fresh complete medium, and 48 h after transfection, cells were used either for Ca^2+^ imaging or fixed for confocal imaging. For Ca^2+^ imaging, transfected cells were mounted in an acquisition chamber and placed on the stage of an epifluorescence Leica DMI6000B microscope equipped with an S Fluor 40×/1.3 objective, a Polychrome V monochromator (Till Photonics), and a cooled CCD camera (Hamamatsu Photonics). An internal lens with a 1.6 optical increment was used. Fluorescence, acquired at 1 s intervals, using a 520/20 bandpass emission filter, was registered using MetaFluor software (Molecular Devices). After the baseline fluorescence was registered, ATP (100 μM) was applied for the rest of the acquisition. For quantification of Ca^2+^ transients, fluorescence at the peak of the response was normalized to the baseline level just prior to ATP addition using a formula Δ*F*/*F*_0_. For confocal imaging, cells were fixed in 4% formaldehyde (20 min, RT), washed three times with D-PBS, and mounted on microscope slides. Images were acquired using Leica SP8 Lightening LCSM and postprocessed using ImageJ.

## Results and Discussion

### Development of Three Self-Complementing Bipartite Fluorescent Proteins

Starting from the *Aequorea*-derived EGFP^29^ variant from iGluSnFR^33^, lancelet-derived NG^31^ from mNG-GECO1 (ref^24^), and *Discosoma*-derived RFP^30^ from R-GECO1 (ref^22^), we developed a series of self-complementing bipartite FPs with the FP9-6 plus FP7-8 topology (Figure 1b). The small fragment (FP7-8) was always expressed as a fusion with the maltose-binding protein (MBP) to enhance the solubility. In each case, directed evolution was used to improve the folding and brightness of the initial prototype construct.

To develop self-complementing bipartite green and red cpFPs, we started with the cpGFP and cpRFP with topology N-7-8-9-10-11-cplinker-1-2-3-4-5-6-C (Figures S1–S3). To mimic a bipartite protein using a single polypeptide chain, a previously reported 32-amino-acid spacer^10^ was inserted between the eighth and ninth strands to give N-7-8-spacer-9-10-11-cplinker-1-2-3-4-5-6-C. Spacer-inserted cpGFP (scpGFP0.1) was dimly fluorescent, and spacer-inserted cpRFP (scpRFP0.1) showed no detectable fluorescence on LB plates. To improve the fluorescence brightness, we performed iterative directed evolution on both spacer-inserted cpFPs. In each round, genetic diversity was created using error-prone PCR to introduce random mutations over the full length of the gene. The resulting library was screened in the context of *Escherichia coli* colonies, and the brightest variant was used as the template for the next round of library creation. Substantially brighter variants were developed using three rounds of evolution for scpGFP (with spacer) and eight rounds for scpRFP (with spacer). These variants were designated as GFP7-8 + GFP9-6 = scpGFP1 (Figure 2a–d) and RFP7-8 + RFP9-6 = scpRFP1 (Figure 2e–h), respectively.

To develop a self-complementing bipartite NG, we first generated a cp version of NG using a previously reported cp linker from an NG-based biosensor.^34^ We then split the gene and cloned NG7-8 and NG9-6 after two different ribosome binding sites (RBS) in a bicistronic bacterial expression plasmid (pBiC) (Figure S4).^35^ Expression of the two polypeptides (scpNG0.1) in *E. coli* resulted in bacterial colonies that were dimly fluorescent. As described above, we performed four rounds of directed evolution (with random mutation of both fragments and the second RBS) and ultimately obtained NG7-8 + NG9-6 = scpNG1 (Figure 2i–l).

To quantitatively evaluate the brightness of the evolved variants, we cloned the GFP7-8 and GFP9-6 fragments and the RFP7-8 and RFP9-6 fragments into the pBiC plasmid under two different RBSs, identical to the scpNG1 expression system. When expressed as separate polypeptides in *E. coli* as colonies on LB plates, scpGFP1, scpRFP1, and scpNG1 all gave comparable or improved brightness (Figure 2c,g,k and Table S1) relative to their nonbipartite full-length parental counterparts (cpGFP, cpRFP, and cpNG, respectively).

We also created blue, cyan, and yellow versions of scpGFP1 by introducing mutations that have been previously reported to produce color variants of GFP (Figure S5 and Table S2). Introducing Thr165Ser and Tyr166His (Thr65Ser and Tyr66His in GFP numbering) mutations produced a blue-shifting variant (scpBFP) with an emission peak at 429 nm. Introducing the Tyr166Trp (Tyr66Trp in GFP numbering) mutation produced a cyan-shifting variant (scpCFP) with an emission peak at 487 nm. Introducing Thr165Gly and Thr58Tyr (Thr65Gly and Thr203Tyr in GFP numbering) produced a yellow-shifting variant (scpYFP) with an emission peak at 521 nm.

### Characterization of Self-Complementing Bipartite Fluorescent Proteins

To characterize each of the newly developed scpFPs, we expressed both polypeptides from a single pBiC plasmid in bacteria and purified the intact self-complemented scpFPs with Ni-NTA affinity chromatography. All scpFPs showed similar spectral properties to their parental cpFPs (Figure 3a and Table S1). Combinations of fragments from different scaffolds did not show any fluorescence above the autofluorescence background, indicating that these scpFPs are orthogonal (Figure 3b). The substantially higher brightness of scpNG1, relative to scpGFP1 (images acquired with same filters and conditions), is consistent with the spectral properties measured with purified proteins (Table S1).

To determine the pH dependence of the bipartite FPs, we diluted the purified protein into a series of pH buffers ranging from 3.0 to 11.5 and measured the fluorescence. Fluorescence intensities were plotted against pH and fit with a sigmoidal curve, revealing that scpGFP1, scpRFP1, and scpNG1 have apparent p*K*_a_ values of 7.0, 5.8, and 7.5, respectively (Figure 3c,f,i). scpNG1 exhibited pH-dependent quenching of fluorescence at values above pH 9, which could be due to ionization of side chains in the chromophore environment or pH-dependent dissociation of the small fragment.

We next asked whether the split FPs could retain their self-complementing ability when expressed in mammalian cells. We transferred the genes of FP7-8 and FP9-6 to a pcDNA3.1­(+) plasmid with the P2A self-cleaving tag^40^ to separate each fragment after translation. When plasmids encoding scpGFP1, scpRFP1, and scpNG1 were used to transiently transfect HeLa cells, we observed the expected localization of bright fluorescence in the cytosol and nucleus (Figure 3d,g,j). The brightness in HeLa cells was compared to those of other available FP1-10 + FP11 systems: Split GFP^7^ for scpGFP1, and mNG3A^11^ for scpNG1 and sfCherry3C^12^ for scpRFP1. scpGFP1 showed 33% of the brightness of split GFP; scpRFP1 exhibited 122% of the brightness of sfCherry3C; and scpNG1 exhibited 180% of the brightness of mNG3A (Figure 3e,h,k). When we expressed the small and large fragments of scpGFP1, scpNG1, and scpRFP1 from the different plasmids, scpGFP1 and scpNG1 showed bright fluorescence in the cytosol. On the other hand, scpRFP1 did not show fluorescence, which could indicate that scpRFP1 is not fully self-complementable when two fragments are apart in living cells (Figure S6a–f).

We next investigated whether the split FPs could be reconstituted in mammalian cells when the small fragment was fused to a protein or organelle of interest and the large fragment was expressed as a soluble cytosolic protein. We constructed several fusions of FP7-8 and organelle-localizing proteins, including calnexin (CNX), actin, TOM20, keratin, vimentin, histone 2B (H2B), and laminB1. In all cases of scpGFP1 and scpNG1, expected localization patterns were observed (Figure S6g–t). In the case of scpRFP1, only CNX and actin patterns were observed, and other fusions did not produce any fluorescence (Figure S6u,v). These observations also support the poor ability of scpRFP1 to self-complement in cells.

### Development of Three Self-Complementing Bipartite Ca^2+^ Biosensors

Starting from scpGFP1, scpNG1, and scpRFP1, we constructed self-complementing bipartite Ca^2+^ biosensors that we designated as split green, neon-green, and red fluorescent genetically encoded Ca^2+^ biosensors for optical imaging 1 (sG-GECO1, sN-GECO1, and sR-GECO1, respectively). The basic design of these biosensors is to have a calmodulin (CaM) binding peptide (CBP), and CaM, fused to the N- and C-termini, respectively, of the FP7-8 small fragment (Figure 1b). The rationale for this design is that the N-terminus of β-strand 7 and the C-terminus of β-strand 8 are in close proximity to each other and, critically, are close to the position of the key permutation site of GCaMP and all other single FP-based Ca^2+^ biosensors.^18^ Indeed, the first residues of β-strand 7 in these constructs are 147 in scpGFP1, 137 in scpNG1, and 146 in scpRFP1. These residues are all within the critical “bulge and gatepost” region of the corresponding FPs, which represents the position where the chromophore most closely approaches the surrounding β-barrel. Essentially, all previously single FP-based biosensors were constructed by inserting responsive domains (e.g., domains that undergo a conformational change upon binding a specific ion or molecule) into this region of the protein. We rationalized that by inducing Ca^2+^-dependent conformational changes in the region around the N-terminus of β-strand 7, and the C-terminus of β-strand 8, the chromophore environment would be modulated such that changes in the fluorescence intensity were likely to be achieved.

When we fused FP7-8 to CaM and CBP, and coexpressed with the large fragments, the resulting proteins exhibited Ca^2+^-dependent Δ*F*/*F*_min_ (= (*F*_max_ – *F*_min_)/*F*_min_) of 0.22 for sG-GECO, 0.09 for sN-GECO, and 0.32 for sR-GECO. To improve the fluorescent response and brightness of all three biosensors, we employed a combination of linker optimization (i.e., the CBP to N-terminus linker and the C-terminus to CaM linker) and directed evolution (Figure S7). Directed evolution was performed by first randomly mutating the whole region of the plasmid encoding the two polypeptide chains and the ribosome binding site (RBS) for the second fragment (i.e., 5′-CBP-FP7-8-CaM-RBS-cpFP9-6). The library of randomly mutated genes was expressed in *E. coli*, brightly fluorescent colonies were picked (approximately 400 per round), clones were cultured overnight in 96-deep-well plates, bacteria were lysed and proteins extracted using a commercial detergent-based reagent, and the Ca^2+^-dependent Δ*F*/*F*_min_ values were determined in 96-well optical bottom plates. Plasmids encoding variants with particularly desirable features (e.g., large Δ*F*/*F*_min_ or high brightness) were isolated by miniprep from the bacterial cell debris pellet (following detergent-based extraction and centrifugation) in the deep-well plate. The plasmid encoding the most promising “winning” variant, or a mixture of several plasmids encoding promising variants, was used as the template for the next round of directed evolution. To produce sN-GECO1, a final round of gene shuffling with four promising variants was performed using the staggered extension process.^36^ This directed evolution procedure was repeated for multiple rounds for each variant, ultimately producing sG-GECO1, sN-GECO1, and sR-GECO1 (Figure 4).

**Figure 4:**
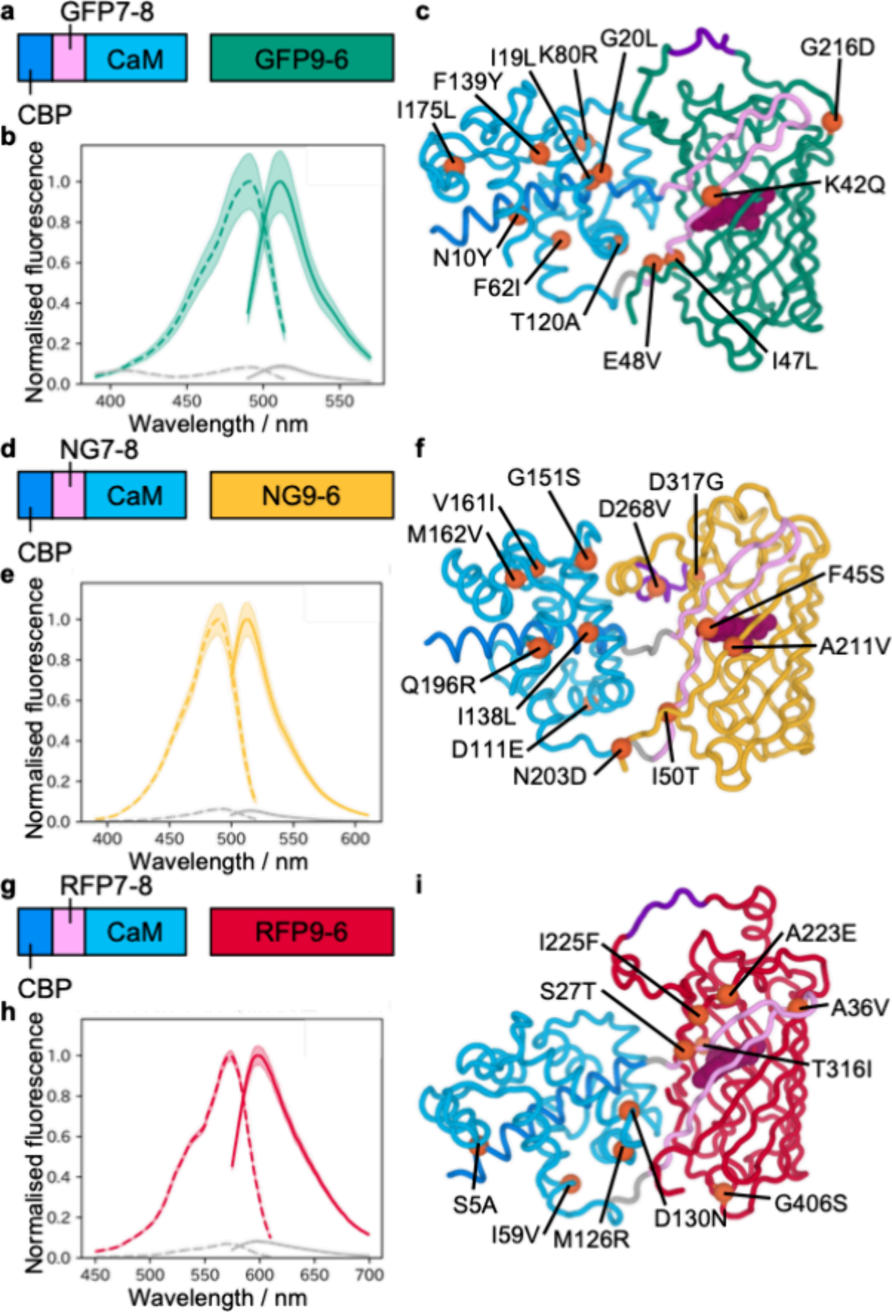
A series of three bipartite Ca^2+^ biosensors. (a) Design of sG-GECO1. CBP, GFP7-8, CaM, and GFP9-6 are highlighted in dark blue, pink, light blue, and green, respectively. Note that sG-GECO1 was expressed as two polypeptides from a pBiC plasmid. (b) Excitation (dashed line) and emission (solid line) spectra of purified sG-GECO1 with (green) and without (gray) 39 μM Ca^2+^. sG-GECO1 has Δ*F*/*F*_min_ (= (*F*_max_ – *F*_min_)/*F*_min_) of 10.5 ± 1.9 with 39 μM Ca^2+^. (c) Structural prediction of sG-GECO1 using AlphaFold-Multimer.^41,42^ The positions of mutations acquired during directed evolution are shown as orange spheres and labeled. The cp linker is colored in purple. The chromophore (dark purple) was positioned based on superposition with cpGFP (PDB ID: 3EVP).^43^ CBP, GFP7-8, CaM, and GFP9-6 are colored in the same way as described in (a). (d) Design of sN-GECO1. CBP, NG7-8, CaM, and NG9-6 are highlighted in dark blue, pink, light blue, and yellow, respectively. (e) Excitation (dashed line) and emission (solid line) spectra of purified sN-GECO1 with (yellow) and without (gray) 39 μM Ca^2+^. sN-GECO1 has Δ*F*/*F*_min_ of 17.5 ± 2.4 with 39 μM Ca^2+^. (f) Structural prediction of sN-GECO1 using AlphaFold-Multimer.^41,42^ The positions of mutations acquired during directed evolution are shown as orange spheres and labeled. The cp linker is colored in purple. The chromophore (dark purple) was positioned based on superposition with mNeonGreen (PDB ID: 5LTR).^44^ CBP, NG7-8, CaM, and NG9-6 are colored in the same way as described in (d). (g) Design of sR-GECO1. CBP, RFP7-8, CaM, and RFP9-6 are highlighted in dark blue, pink, light blue, and red, respectively. (h) Excitation (dashed line) and emission (solid line) spectra of purified sR-GECO1 with (red) and without (gray) 39 μM Ca^2+^. sR-GECO1 has Δ*F*/*F*_min_ of 11.2 ± 0.5 with 39 μM Ca^2+^. (i) Structural prediction of sR-GECO1 using AlphaFold-Multimer.^41,42^ The positions of mutations acquired during directed evolution are shown as orange spheres and labeled. The cp linker is colored in purple. The chromophore (dark purple) was positioned based on superposition with mCherry (PDB ID: 2H5Q).^45^ CBP, RFP7-8, CaM, and RFP9-6 are colored in the same way as described in (g).

### *In Vitro* Characterization of Three Bipartite Ca^2+^ Biosensors

To characterize each of the newly developed bipartite Ca^2+^ biosensors, we expressed both polypeptides separately from a pBiC plasmid and purified them with Ni-NTA affinity chromatography. All bipartite Ca^2+^ biosensors showed similar spectral properties to their parental scpFPs (Tables S1 and S3). Purified sG-GECO1, sN-GECO1, and sR-GECO1 exhibited Δ*F*/*F*_min_ values of 10.5 ± 1.9, 17.5 ± 2.4, and 11.2 ± 0.5 with 39 μM Ca^2+^, respectively (Figure 4).

Purified sG-GECO1 has absorbance peaks at 395 and 491 nm, which are attributed to the neutral (protonated) and anionic (deprotonated) forms of the chromophore, respectively (Figure 5a). Similarly, purified sR-GECO1 has absorbance peaks at 448 and 575 nm, which are also attributed to the neutral and anionic forms of the chromophore, respectively (Figure 5b). Absorbance spectra of sG-GECO1 and sR-GECO1 revealed that their Ca^2+^-dependent fluorescence changes come from population changes of nonfluorescent neutral chromophore fraction and fluorescent anionic chromophore fraction. In contrast, the absorbance spectra of sN-GECO1 did not provide support for such a Ca^2+^-dependent population change (Figure 5c). This suggests that an alternative mechanism may be responsible for the fluorescence change. The absorbance peaks of sN-GECO1 were at 462 nm in the absence of Ca^2+^ and 488 nm in the presence of Ca^2+^.

**Figure 5:**
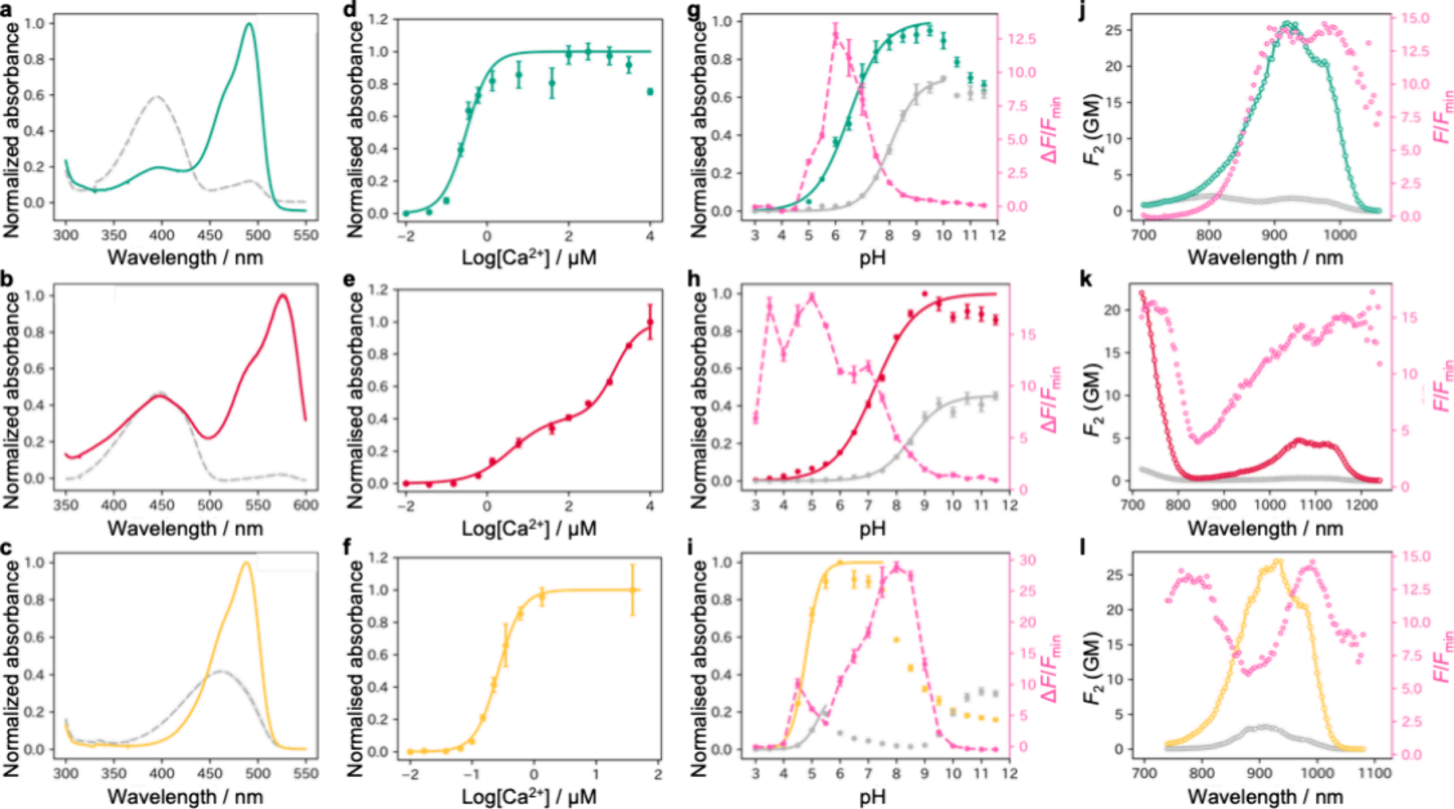
*In vitro* characterization of bipartite Ca^2+^ biosensors. Absorbance spectra of (a) sG-GECO1, (b) sR-GECO1, and (c) sN-GECO1 with and without (gray) 39 μM Ca^2+^. Ca^2+^ titration curve of (d) sG-GECO1, (e) sR-GECO1, and (f) sN-GECO1. Data are shown as the mean ± standard deviation (*n* = 3). pH titration curve of (g) sG-GECO1, (h) sR-GECO1, and (i) sN-GECO1 with and without (gray) Ca^2+^. For sG-GECO1 and sN-GECO1, 39 μM Ca^2+^ was used. For sR-GECO1, 10 mM Ca^2+^ was used. We were unable to reliably measure a p*K*_a_ of sN-GECO1 in the absence of Ca^2+^. Calculated Δ*F*/*F*_min_ is shown in pink. Data are shown as mean ± standard deviation (*n* = 3). Two-photon excitation spectra of (j) sG-GECO1, (k) sR-GECO1, and (l) sN-GECO1 with and without (gray) 39 μM Ca^2+^. Calculated Δ*F*/*F*_min_ is shown in pink.

We next carried out Ca^2+^ titrations, which revealed that sG-GECO1 exhibit monophasic dose–response curves and have apparent dissociation constants to Ca^2+^ (*K*_d,app_) of 306 nM (Figure 5d and Table S3). sR-GECO1 has a biphasic dose–response curve consistent with *K*_d,app_ values of 3.85 μM and 1.34 mM (Figure 5e and Table S3). The wide dynamic range of sR-GECO1 (biphasic curve with two Hill coefficients of 0.92 and 1.42) might be useful in systems where the concentration of Ca^2+^ varies over a wide range. sN-GECO1 shows a *K*_d,app_ of 268 nM (Figure 5f and Table S3).

To check their pH dependence, we diluted the purified proteins into a series of pH buffers ranging from 3.0 to 11.5 either with or without Ca^2+^, and we measured the fluorescence. Fluorescence intensities were plotted against pH with the sigmoidal curve. sG-GECO1 and sN-GECO1 have p*K*_a_ values of 6.5 and 4.8, respectively, in the presence of 39 μM Ca^2+^, while sR-GECO1 has a p*K*_a_ of 7.2 in the presence of 10 mM Ca^2+^ (Figure 5g–i and Table S3).

The two-photon excitation maximum of Ca^2+^-bound sG-GECO1 is 920 nm with a brightness of *F*_2_ = 26 GM and a two-photon excited fluorescence change (Δ*F*_2_/*F*_2_) of 14 (Figure 5j and Table S4). The peak two-photon absorption cross sections of the sG-GECO1 anionic chromophore (32 ± 5 and 36 ± 6 GM for Ca^2+^-saturated and free states, respectively) match within experimental errors the previously reported value of the parent EGFP (39 ± 8 GM).^46^ The two-photon excitation spectra display two overlapping peaks within the absorption region of the anionic chromophore (Figure 5j–l). We attribute the peak at the longer wavelength to the pure electronic (0–0) transition and the peak at the shorter wavelength to a vibronic (0–1) transition of the anionic chromophore. Notably, unlike in one-photon absorption spectra, the vibronic peak exhibits a higher intensity than that of the pure electronic peak. This phenomenon has been previously attributed to an unusual vibronic coupling specifically characteristic of two-photon absorption spectra.^47^ The two-photon brightness of Ca^2+^-bound sR-GECO1 excited at 1072 nm, *F*_2_ = 4.5 GM, is comparable to that of Ca^2+^-bound R-GECO1 (*F*_2_ = 5 GM) (Table S4). sN-GECO1 also shows a Ca^2+^-induced two-photon-excited fluorescence change (Δ*F*_2_/*F*_2_ = 8.3) at 928 nm (Figure 5l). The two-photon cross section of sN-GECO1 anionic chromophore of the Ca^2+^-saturated state (41 ± 5 GM) is significantly larger than that of its parent protein mNeonGreen (29 ± 4 GM).^48^ We attribute this observation to a blue shift of the one-photon absorption peak of sN-GECO1 (488 nm) compared to mNeonGreen (506 nm), which according to our physical model should result in an increase of the two-photon absorption.^48^ From the structural point of view, the blue shift of the sN-GECO1 anionic peak in the Ca^2+^-saturated state signals the distortion of the local chromophore environment upon genetic modifications introduced to create sN-GECO1.

To gain further insight into the structure and mechanism of sG-GECO1, we attempted to determine its atomic structure by X-ray crystallography (Figure S8 and Table S5). Although we succeeded in growing diffraction-quality crystals, we were able to obtain only a partial structure. In this partial structure, the electron density was observed for the FP domain but not for the CaM and CBP domains.

### Application of the Bipartite Ca^2+^ Biosensor at the ER-Mitochondria Interface

Finally, we applied sN-GECO1 for monitoring the dynamics of the Ca^2+^ concentration at the ER-mitochondria interface. We first tested the exact ER-mitochondria distance at which the complementation of split NG occurs (Figure S9) and then designed an all-in-one construct to ensure equimolar expression^49–51^ of the sN-GECO1 fragments targeted to the membranes of mitochondria and ER, respectively, using minimal targeting sequences (Figure 6a). HeLa cells expressing this construct, designated SPLICa:ER-MT, displayed a punctate fluorescence pattern that colocalized well with the fluorescence markers of endogenous ER and mitochondria (Figure 6b). This result suggests that sN-GECO1-based SPLICa:ER-MT localizes at MERCs. Notably, parallel attempts with sG-GECO1 and sR-GECO1 did not yield observable fluorescence, highlighting their limitations in mammalian cell applications at MERCs.

**Figure 6:**
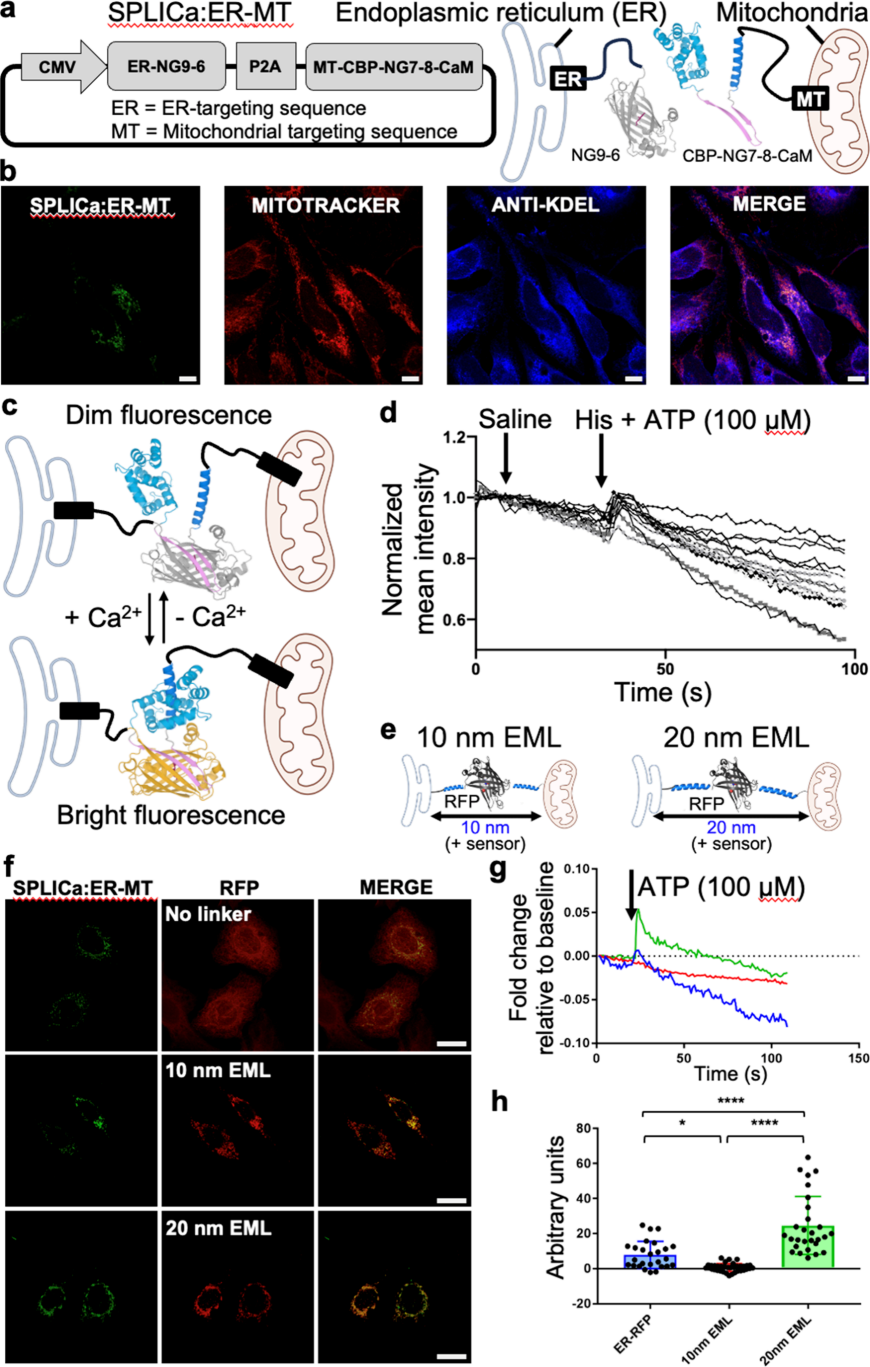
Imaging of Ca^2+^ dynamics changes at the ER-mitochondria interface. (a) Schematic representation of the SPLICa:ER-MT vector based on sN-GECO1 and the protein components at the ER–mitochondria contact sites. The NG9-6 fragment, targeted to the ER surface, is cloned upstream of the P2A self-cleaving peptide, while the downstream cassette encodes the mitochondrial-surface-targeted CBP-NG7-8-CaM fragment. (b) Confocal images of HeLa cells transfected with the SPLICa:ER-MT construct (green). Labeled with MitoTracker Red CMXRos for mitochondria (red). Immunostained with anti-KDEL antibody for the ER (blue). Scale bars, 10 μm. (c) Schematic illustration of Ca^2+^-dependent modulation of the SPLICa:ER-MT signal. Upon elevation of Ca^2+^ levels at the ER-mitochondria contact sites, the probe increases fluorescence brightness. (d) Representative fluorescence traces showing the dynamic response of SPLICa:ER-MT to ATP/histamine stimulation (100 μM) in HeLa cells. (e) Schematic illustration of using EMLs to maintain the ER-mitochondrial distances at either 10 nm (10 nm EML) or 20 nm (20 nm EML). (f) Confocal images of HeLa cells cotransfected with SPLICa:ER-MT (green) and either ER-RFP (no linker), 10 nm EML, or 20 nm EML (all in red). Scale bars, 20 μm. (g) Representative fluorescent traces of SPLICa:ER-MT dynamics in response to ATP stimulation (100 μM) in cells coexpressing ER-RFP (control, no linker, blue), 10 nm EML (red), or 20 nm EML (green). (h) Quantification of the fluorescence response of SPLICa:ER-MT upon ATP stimulation. Data are expressed as mean ± standard error of mean (SEM) normalized fluorescence from 26 to 36 cells in three independent experiments (three to five coverslips per experiment). One-way ANOVA with Tukey posthoc test. *p = 0.022; ****p < 0.0001.

Adenosine triphosphate (ATP)^52,53^ and histamine^54^ work as agonists to the inositol 1,4,5-triphosphate (IP_3_) receptor, inducing Ca^2+^ transfer from ER to mitochondria.^55^ To visualize the Ca^2+^ dynamics at the ER-mitochondria contact sites, we applied these agonists to SPLICa:ER-MT expressing HeLa cells. This imaging resulted in the increase in fluorescence intensity of SPLICa:ER-MT upon the agonists, while saline did not induce a substantial change of fluorescence intensity (Figure 6c,d). These results indicate that SPLICa:ER-MT enables monitoring of Ca^2+^ transfer between the ER and mitochondria in living cells.

IP_3_ receptor-mediated Ca^2+^ transfer at the ER-mitochondria interface requires a highly specific distance.^38^ To test the sensitivity of SPLICa:ER-MT to subtle variations in the concentration of Ca^2+^ as a function of distance between ER and mitochondria, we employed synthetic ER-mitochondrial linkers (EMLs) that precisely maintain the ER-outer mitochondrial membrane (OMM) distance at 10 and 20 nm—the suboptimal and optimal distances for efficient Ca^2+^ transfer, respectively (Figure 6e,f).^38^ We estimate the biosensor’s intrinsic contribution to the ER-mitochondria gap to be approximately 4 nm, based on its AlphaFold structural model (Figure S10). Therefore, the effective distances analyzed are approximately 14 and 24 nm when using the 10 and 20 nm EML constructs, respectively. The difference in fluorescence patterns (fusiform vs circular) under the 10 and 20 nm EML conditions may arise from variability in cell morphology or from alterations in ER–mitochondria contact organization induced by the rigid structure and spacer length of the EML constructs.^55^ When the 20 nm EML was coexpressed with SPLICa:ER-MT, a pronounced increase in the fluorescence peak was observed (green trace) compared to control cells expressing SPLICa:ER-MT alone (blue trace) (Figure 6g,h), indicating high sensitivity to Ca^2+^ changes at the ER-mitochondria interface as a function of distance. Interestingly, when the suboptimal 10 nm EML was coexpressed with SPLICa:ER-MT, the Ca^2+^ response to stimulation with IP_3_-linked agonists was strongly affected (red trace) (Figure 6g,h). This highlights the ability of SPLICa:ER-MT to detect subtle variations in the ER-mitochondria Ca^2+^ microdomain depending on interorganelle spacing.

## Conclusions

In this study, we report the development of self-complementing bipartite fluorescent proteins (scpFPs), namely, scpGFP1, scpNG1, and scpRFP1, derived from the full-length FPs EGFP, mNeonGreen (NG), and mApple RFP, respectively. These proteins were engineered by dividing the original proteins into two nonfluorescent fragments: one fragment comprising β-strands 7–8 (FP7-8) and the other fragment consisting of β-strands 9-10-11-1-2-3-4-5-6 (cpFP9-6). Comprehensive characterization both *in vitro* and in living HeLa cells demonstrated that these scpFPs spontaneously reassemble into fluorescent proteins without requiring additional interacting partners.

Utilizing these developed scpFPs, we further engineered a series of self-complementing Ca^2+^ biosensors by genetically fusing the Ca^2+^ binding protein (CaM) and CaM-binding peptide (CBP) to the smaller fragment, FP7-8. Extensive protein engineering, including linker optimization and directed evolution, yielded three biosensors: sG-GECO1, sN-GECO1, and sR-GECO1, exhibiting Δ*F*/*F*_min_ values of 10.5, 17.5, and 11.2, respectively. Cellular assays confirmed that these biosensors reliably functioned as Ca^2+^ indicators under physiological conditions. To test if the split FP-based biosensor enables monitoring of Ca^2+^ dynamics at organelle contact sites, sN-GECO1 was targeted to mitochondria-ER contact sites (MERCs). Live-cell imaging demonstrated that this biosensor, designated SPLICa:ER-MT, effectively detects Ca^2+^ fluctuations in MERCs upon treatment with an IP_3_ agonist. Our imaging analysis reveals substantial variability in Ca^2+^ dynamics at MERCs among individual cells. While the precise source of this variability remains unclear, assuming that intrinsic biosensor performance is consistent, it is plausible that the observed differences reflect cell-to-cell heterogeneity in the spatial organization and functional engagement of ER-mitochondria contacts. Such heterogeneity could carry significant physiological implications, potentially representing diverse metabolic states or distinct functional configurations in individual cells.

Several previous studies have reported the use of split FP-based biosensors for monitoring intracellular signaling activities.^26–28,56,57^ Typically, these biosensors were split between the sixth and seventh β-strands, necessitating the fusion of each fragment to interacting protein partners to induce fluorescence complementation. Thus, previously developed split FP-based biosensors were not inherently self-complementing. In contrast, our approach introduces an innovative topology wherein FPs are split into two distinct fragments—a nine β-strand fragment (FP9-6; N-9-10-11-cplinker-1-2-3-4-5-6-C) and a two β-strand fragment (FP7-8)—allowing spontaneous reconstitution without external interaction partners.

Recently, G-protein-coupled receptors (GPCRs) have emerged as promising scaffolds for FP-based biosensors by inserting cpFPs into their intracellular loops.^58,59^ While our current demonstration utilizes soluble sensing domains (CaM and CBP), the compact FP7-8 fragment could potentially be inserted into GPCR intracellular loops, facilitating the development of novel GPCR-based split fluorescent biosensors. Notably, GPCRs, being membrane-embedded proteins, are typically resistant to conventional splitting approaches; hence, our small FP7-8 fragment represents a valuable advancement. Additionally, the small size of the FP7-8 fragment offers advantages for endogenous protein labeling via genome editing techniques such as knock-in strategies.^1^

We previously reported that the ER-to-mitochondrial Ca^2+^ flux and mitochondrial bioenergetics are critically influenced by the spatial arrangement between these organelles. Specifically, Ca^2+^ transfer is significantly reduced when the ER-mitochondrial distance is ≤10 nm, whereas an optimal transfer occurs at approximately 20 nm.^38^ Although our earlier observations of this phenomenon relied on Ca^2+^ measurements in mitochondrial compartments (matrix, cristae lumen, and intermembrane space), direct evidence of ER Ca^2+^ release at MERCs remained elusive. Utilizing our MERCs-targeted sN-GECO1, SPLICa:ER-MT, we have now provided direct evidence confirming our hypothesis: Modulation of the ER-mitochondria distance primarily impacts IP_3_ receptor-mediated Ca^2+^ release at MERCs.

Looking forward, our biosensor design strategy can potentially be expanded to detect other biologically relevant signaling molecules, such as dopamine, glutamate, and reactive oxygen species, particularly those involved in organelle contact site signaling. Furthermore, this innovative approach could be employed to visualize molecular dynamics at intercellular junctions including synapses. The development of the self-complementing split FP-based biosensors described herein is expected to significantly enhance our understanding of signaling dynamics at organelle interfaces and other cellular contact sites.

## Supplementary Material



## Notes

Sequence alignment of scpGFP1 and sG-GECO1, sequence alignment of scpNG1 and sN-GECO1, sequence alignment of scpRFP1 and sR-GECO1, plasmid map of pBiC plasmids, blue, cyan, and yellow color variants of scpGFP1, characterization of scpFPs expressed in mammalian cells, lineages of the development of the bipartite Ca^2+^ biosensors, attempted determination of the crystal structure of sG-GECO1 in the Ca^2+^-bound state, testing of various linker lengths between the ER-targeting sequence and the NG9-6 fragment, AlphaFold model of sN-GECO1, spectral and biophysical parameters of the split FPs and their corresponding parent FPs, spectral properties of previously reported FP1-10 + FP11 split FPs, color derivatives of scpGFP1, spectral and biophysical parameters of bipartite Ca^2+^ biosensors, one- and two-photon photophysical parameters of bipartite Ca^2+^ biosensors, X-ray data collection and refinement statistics, and experimental methods (PDF)
