# Supplementary material for: Bipartite Genetically Encoded Biosensors to Sense Calcium Ion Dynamics at Membrane–Membrane Contact Sites
Source: Anal Chem. 2025 Sep 1;97(36):19848–61. doi: 10.1021/acs.analchem.5c03831 (PMC12444748; doi:10.1021/acs.analchem.5c03831)
Supplement: Supplementary file 1 [file ac5c03831_si_001.pdf]

SUPPORTING INFORMATION

Bipartite Genetically Encoded Biosensors to Sense Calcium Ion Dynamics at Membrane–Membrane Contact Sites

Issei Yamaguchi[1†], Lucia Barazzuol[2†], Giulia Dematteis[3], Wenchao Zhu[1], Yurong Wen[4], Mikhail Drobizhev[5], Dmitry Lim[3], Robert E. Campbell[1,6,7], Tito Calì[2,8,9]* and Yusuke Nasu[1,10,11]*

[1] Department of Chemistry, School of Science, The University of Tokyo, Bunkyo-ku, Tokyo 113-0033, Japan.

[2] Department of Biomedical Sciences, University of Padova, Padova 35131, Italy.

[3] Department of Pharmaceutical Sciences, Università del Piemonte Orientale, Novara 28100, Italy.

[4] Center for Microbiome Research of Med-X Institute, The First Affiliated Hospital, Xi’an Jiaotong University, Xi’an, Shaanxi 710061, China.

[5] Department of Microbiology and Cell Biology, Montana State University, Bozeman, Montana 59717, USA.

[6] CERVO Brain Research Centre, Québec, Quebec G1J 2G3, Canada.

[7] Department of Biochemistry, Microbiology, and Bio-informatics, Laval University, Québec, Quebec G1V 0A6, Canada.

[8] Padova Neuroscience Center, University of Padova, Padova 35131, Italy.

[9] Study Center for Neurodegeneration, University of Padova, Padova 35131, Italy.

[10] Institute of Biological Chemistry, Academia Sinica, Nankang, Taipei 115, Taiwan.

[11] Institute of Biochemical Sciences, National Taiwan University, Da’an, Taipei 106, Taiwan.

† These authors contributed equally.

*Correspondence to: Tito Calì (tito.cali@unipd.it) and Yusuke Nasu (nasu@as.edu.tw).

Table of Contents

Figure (p. 3–12)	
Figure S1	Sequence alignment of scpGFP1 and sG-GECO1.
Figure S2	Sequence alignment of scpNG1 and sN-GECO1.
Figure S3	Sequence alignment of scpRFP1 and sR-GECO1.
Figure S4	Plasmid map of pBiC plasmids.
Figure S5	Blue, cyan, and yellow color variants of scpGFP1.
Figure S6	Characterization of scpFPs expressed in mammalian cells.
Figure S7	Lineages of the development of the bipartite Ca^{2+} biosensors.
Figure S8	Attempted determination of the crystal structure of sG-GECO1 in the Ca^{2+}-bound state.
Figure S9	Testing of various linker lengths between the ER-targeting sequence and the NG9-6 fragment.
Figure S10	AlphaFold model of sN-GECO1
Table (p. 13–18)	
Table S1	Spectral and biophysical parameters of the split FPs and their corresponding parent FPs.
Table S2	Spectral properties of previously reported FP1-10 + FP11 split FPs, and the color derivatives of scpGFP1.
Table S3	Spectral and biophysical parameters of bipartite Ca^{2+} biosensors.
Table S4	One- and two-photon photophysical parameters of bipartite Ca^{2+} biosensors.
Table S5	X-ray data collection and refinement statistics.
Methods (p. 19–23)	
References (p. 24–26)	

SUPPLEMENTARY FIGURES

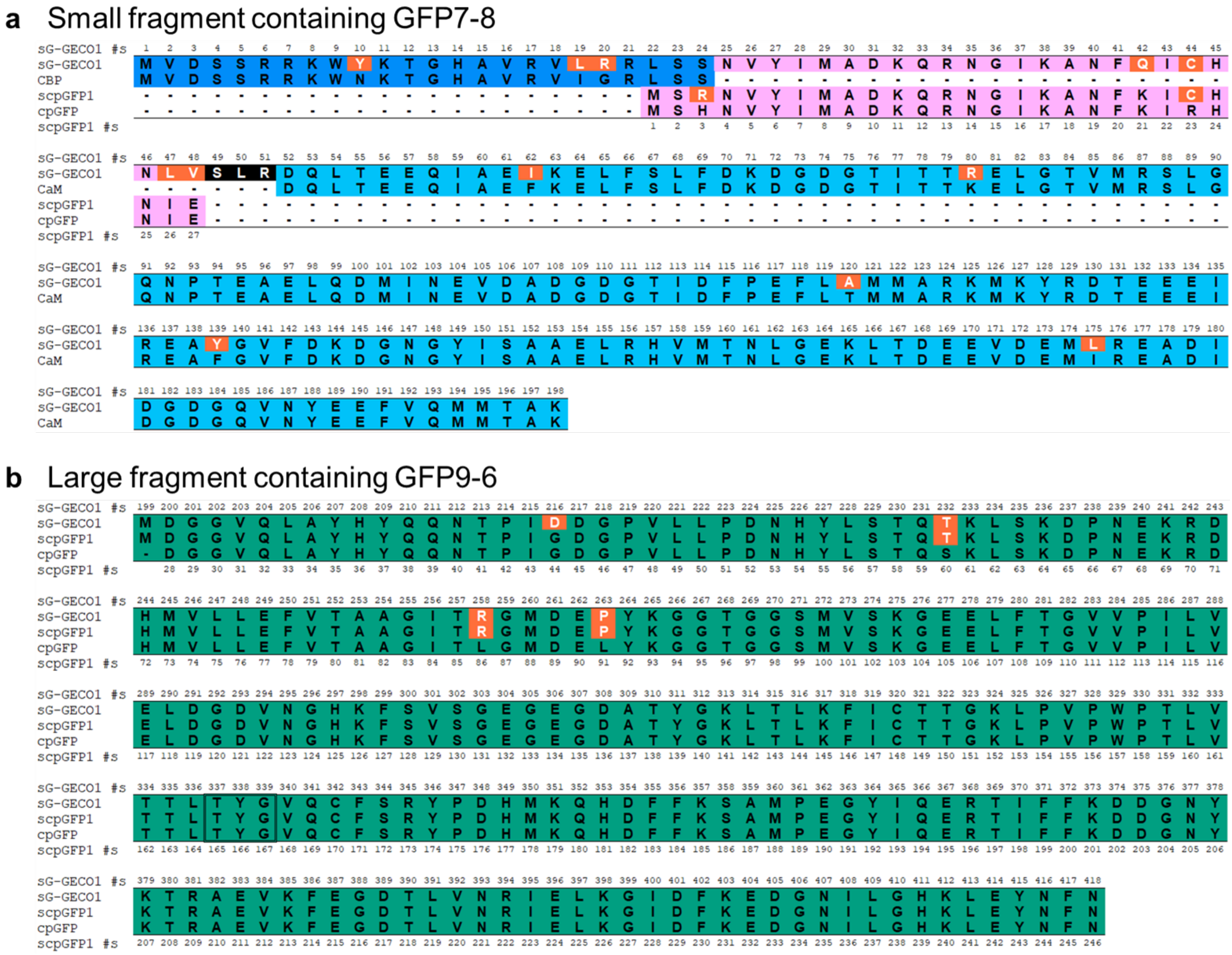

Figure S1 | Sequence alignment of scpGFP1 and sG-GECO1. The GFP7-8 fragment (**a**) and GFP9-6 fragment (**b**) are highlighted in pink and green, respectively. CBP and CaM are highlighted in dark blue and light blue, respectively. Mutations in sG-GECO1 and scpGFP1, relative to their parent proteins, are highlighted in orange. Linker between GFP7-8 and CaM is highlighted in black.

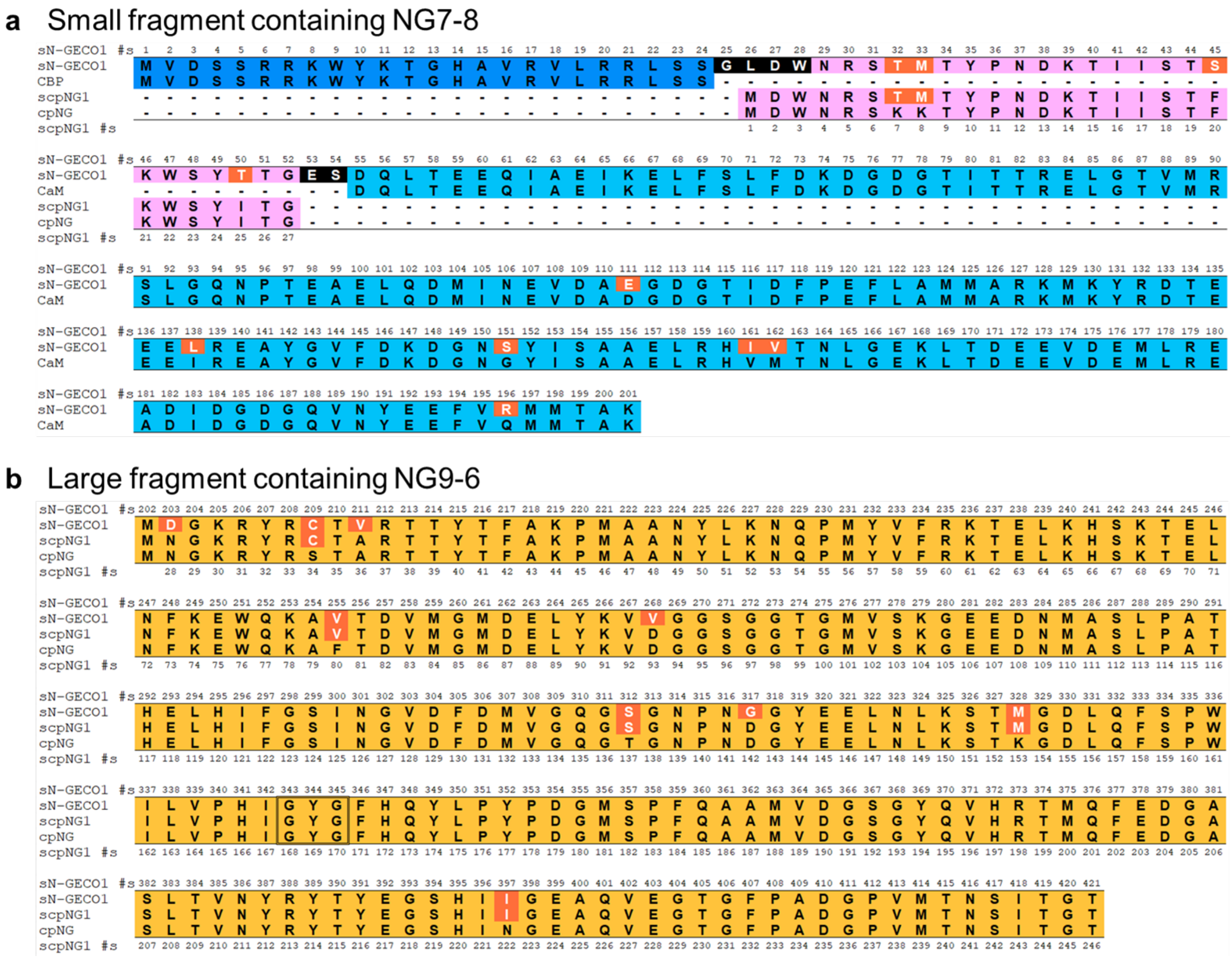

Figure S2 | Sequence alignment of scpNG1 and sN-GECO1. The NG7-8 fragment (**a**) and NG9-6 fragment (**b**) are highlighted in pink and yellow, respectively. CBP and CaM are highlighted in dark blue and light blue, respectively. Mutations in sN-GECO1 and scpNG1, relative to their parent proteins, are highlighted in orange. Linkers between CBP and NG7-8, or NG7-8 and CaM are highlighted in black.

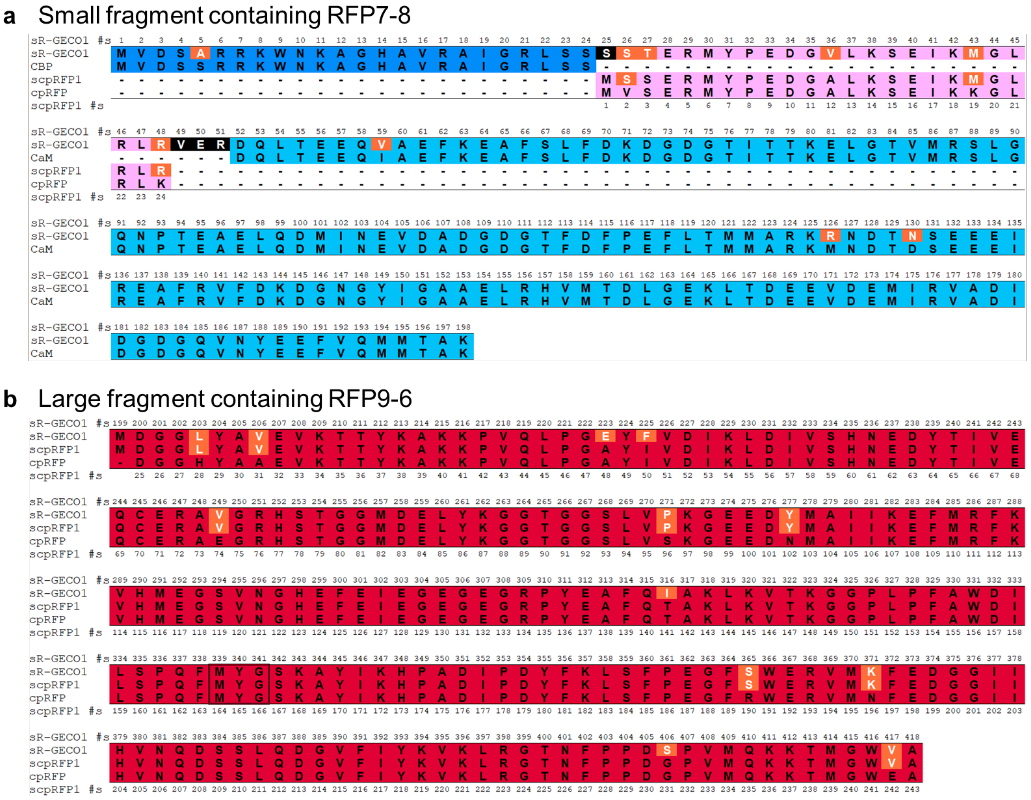

Figure S3 | Sequence alignment of scpRFP1 and sR-GECO1. The RFP7-8 (**a**) and RFP9-6 (**b**) fragments are highlighted in pink and red, respectively. CBP and CaM are highlighted in dark blue and light blue, respectively. Mutations in sR-GECO1 and scpRFP1, relative to their parent proteins, are highlighted in orange. Linkers between CBP and RFP7-8, or RFP7-8 and CaM are highlighted in black.

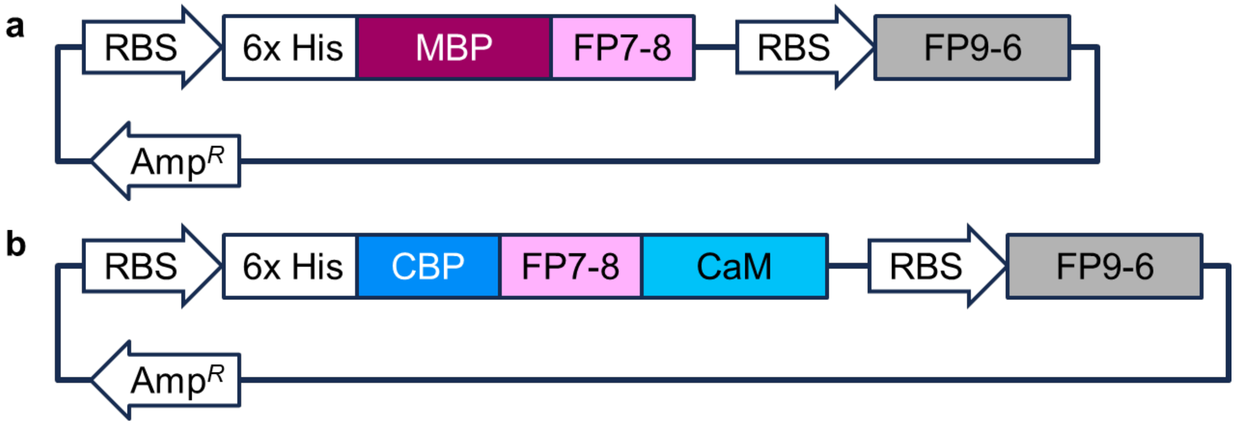

Figure S4 | Plasmid map of pBiC plasmids. pBiC is a plasmid that enables the expression of two different polypeptides under different ribosome binding sites. (**a**) scpFP in the pBiC plasmid. RBS: ribosome binding site, 6× His: hexahistidine tag, MBP: maltose binding protein, FP7-8: small fragment of scpFP consisting of the 7th and 8th strands. FP9-6: large fragment of scpFP consisting of the 9th through 6th strands. Amp^{R}: ampicillin resistance gene. (**b**) Bipartite Ca^{2+} biosensors in the pBic plasmid. CBP: calmodulin binding peptide, CaM: calmodulin.

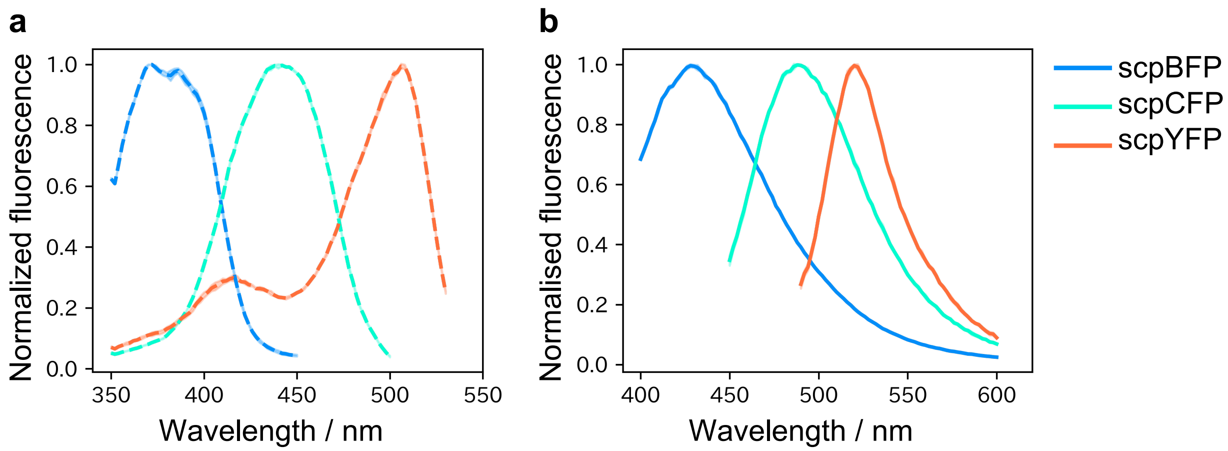

Figure S5 | Blue, cyan, and yellow color variants of scpGFP1. Excitation (**a**) and emission (**b**) spectra of blue, cyan and yellow versions of scpGFP1.

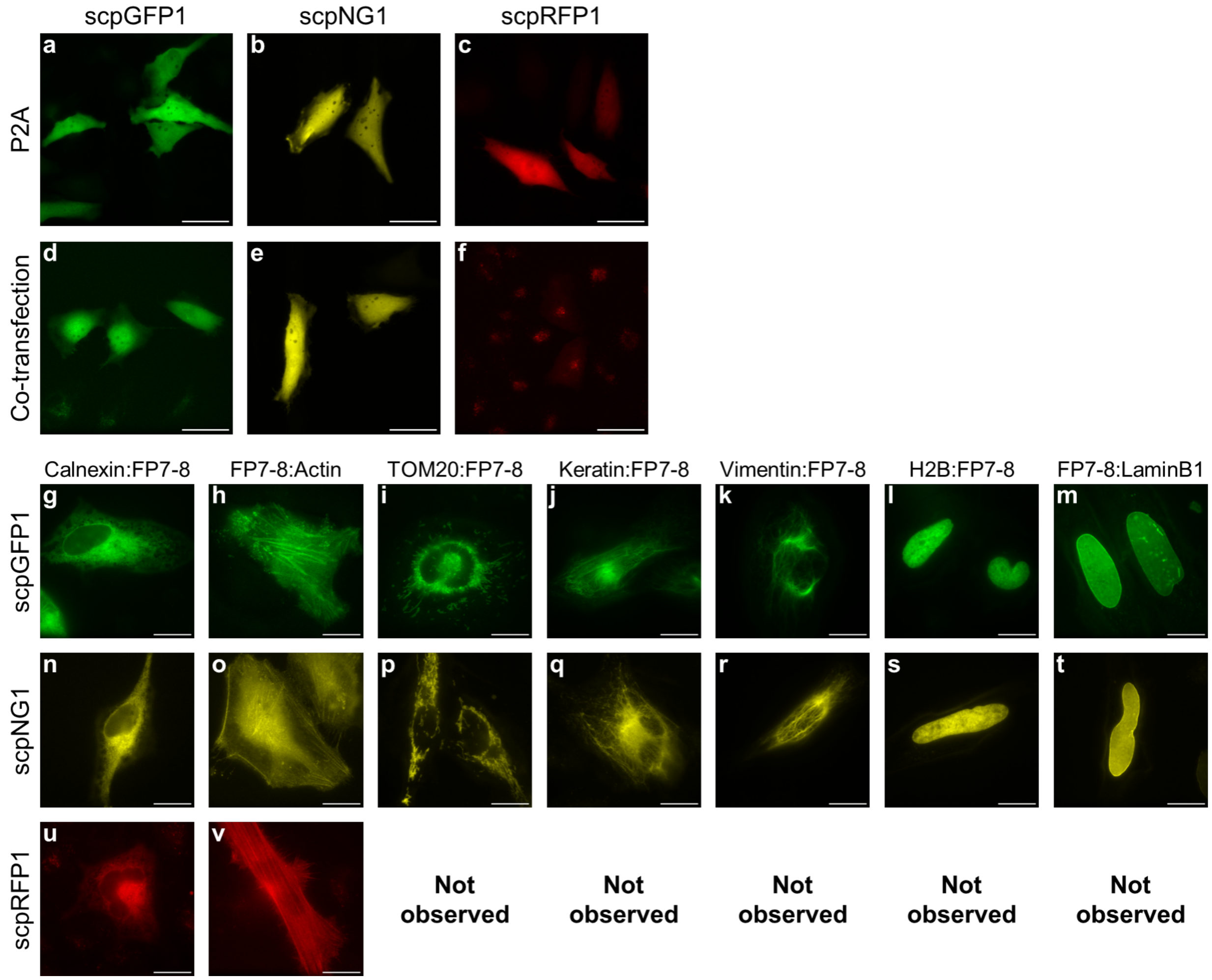

Figure S6 | Characterization of scpFPs expressed in mammalian cells. (**a**–**f**) HeLa cells expressing scpGFP1 (a,d), scpNG1 (b,e), and scpRFP1 (c,f), expressed from a single plasmid via P2A peptide (a–c), or from separate plasmids (d–f). Scale bars are 50 μm. (**g**–**v**) HeLa cells expressing scpGFP1 (g–m), scpNG1 (n–t), or scpRFP1 (u–v), targeting several organelles. A large FP9-6 fragment was expressed in cytosol and a small FP7-8 fragment was expressed in calnexin (CNX) (g,n,u), actin (h,o,v), TOM20 (i,p), keratin (j,q), vimentin (k,r), H2B (l,s) or laminB1 (m,t). scpRFP1 did not show any detectable fluorescence above background autofluorescence with TOM20, keratin, vimentin, H2B and laminB1 fusions (described as 'Not observed'). Scale bars, 20 μm.

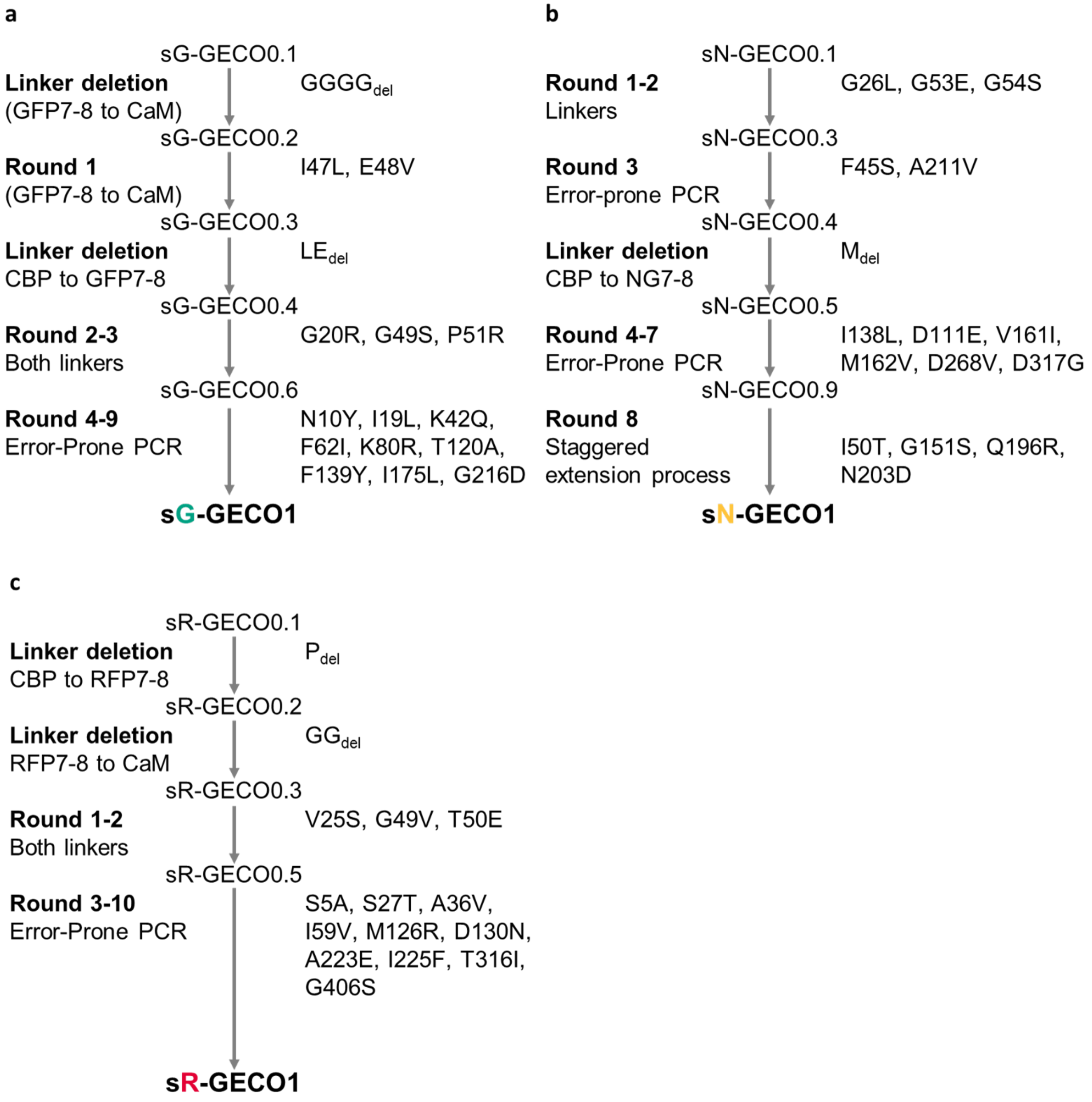

Figure S7 | Lineages of the development of the bipartite Ca^{2+} biosensors. (**a**) scpGFP1. (**b**) scpNG1. (**c**) scpRFP1.

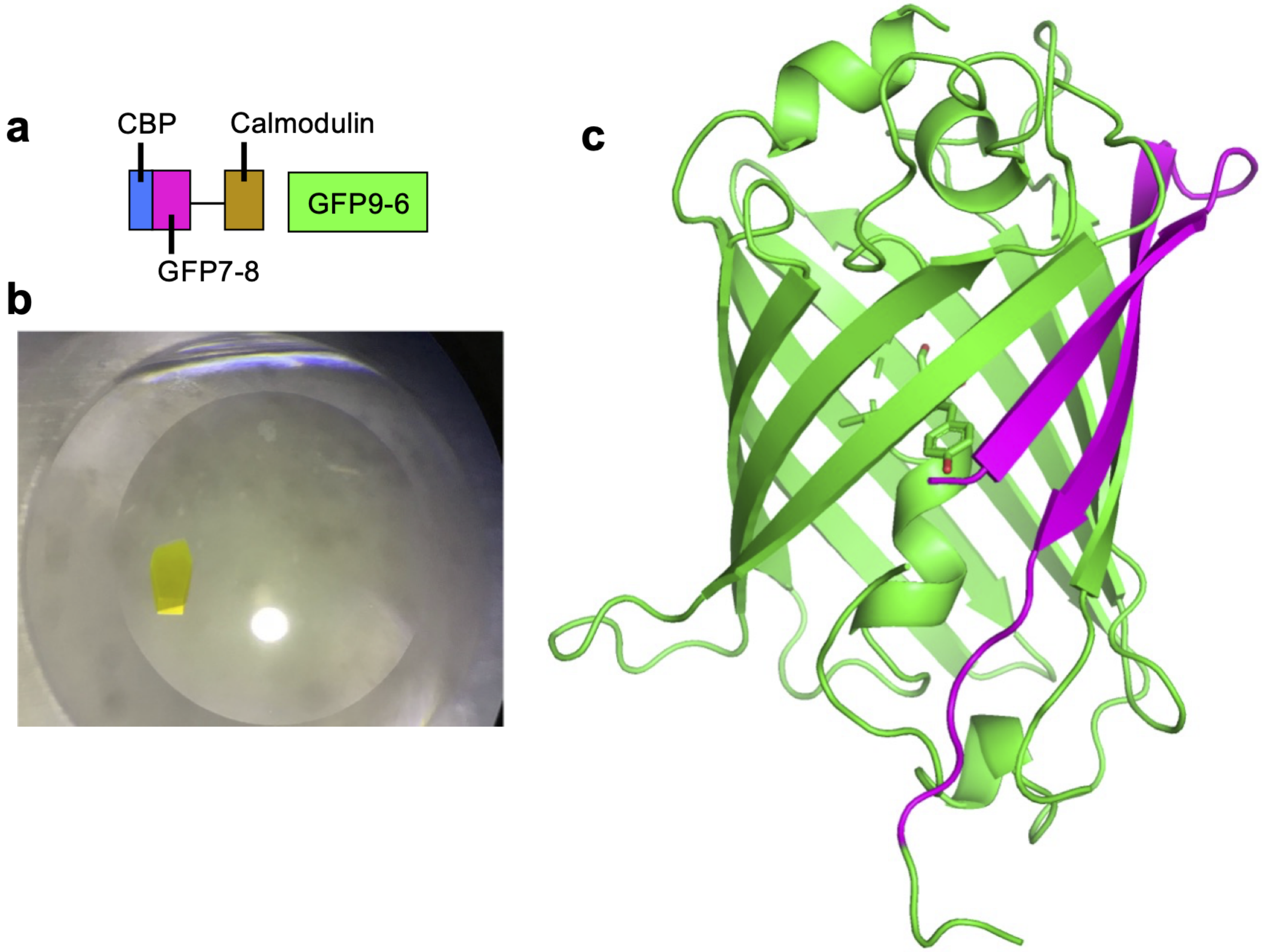

Figure S8 | Attempted determination of the crystal structure of sG-GECO1 in the Ca^{2+}-bound state. (**a**) Representation of the sG-GECO1 protein construct that was purified for crystallization. (**b**) Crystal of sG-GECO1. (**c**) Experimentally determined structure (PDB ID: 9U9D). Electron density corresponding to the CaM and CBP domains was not observed, likely due to their intrinsic flexibility causing variable orientations relative to the rigidly packed GFP domain.

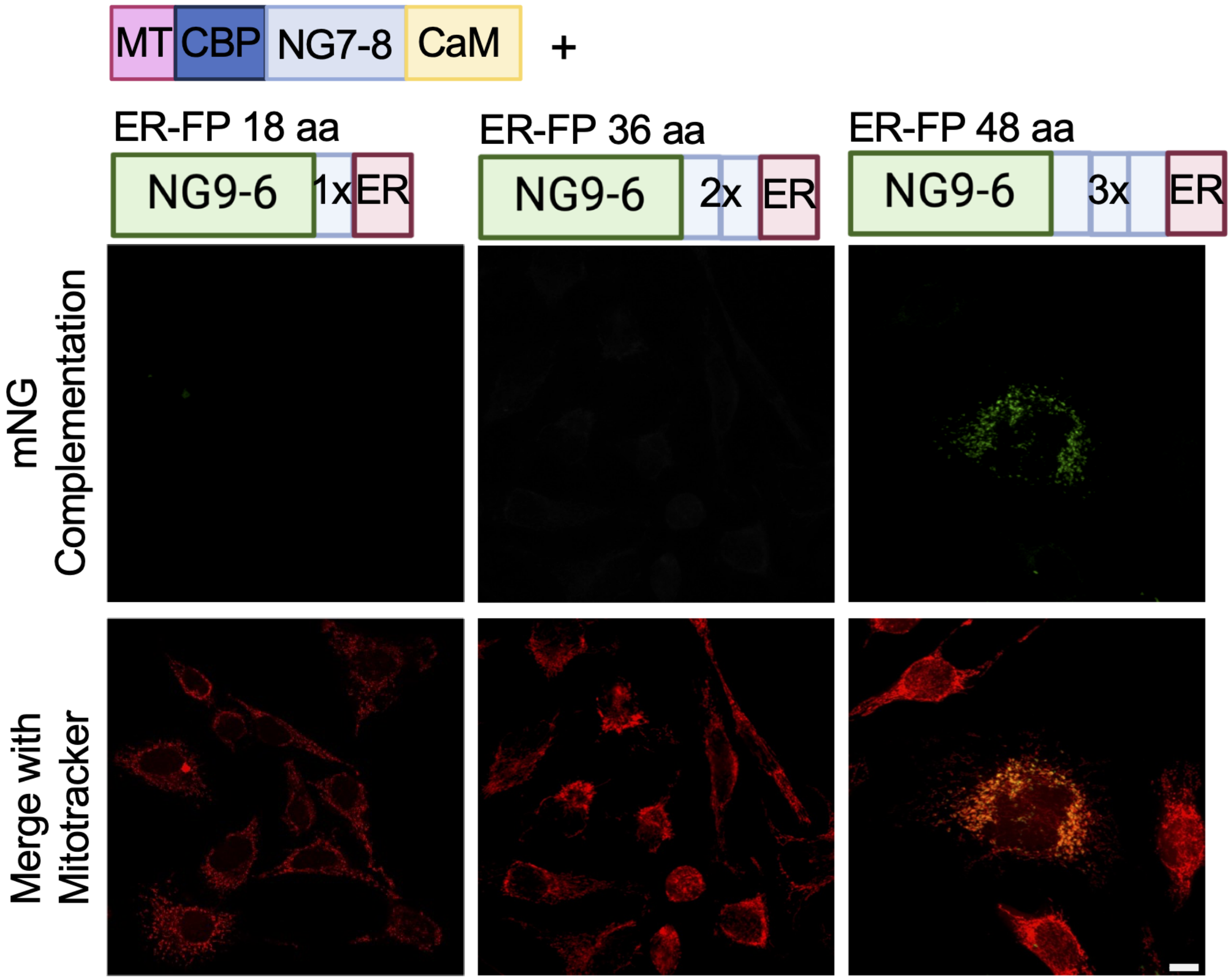

Figure S9 | Testing of various linker lengths between the ER-targeting sequence and the NG9-6 fragment. Schematic representation of the mitochondria targeted SPLICa:ER-MT fragment CBP-NG7-8-CaM and the ER targeted fragment NG9-6 extended in the cytosolic side by 18, 36, or 48 amino acids (top). Functional complementation of the split NG fluorescent fragments overexpressed in HeLa cells occurs exclusively at organelle contact sites when the 48 amino acid spacer is used (bottom). Confocal images of HeLa cells transfected with the combination of the above shown constructs are reported, along with the mitochondrial staining by Mitotracker. Scale bar, 10 μm.

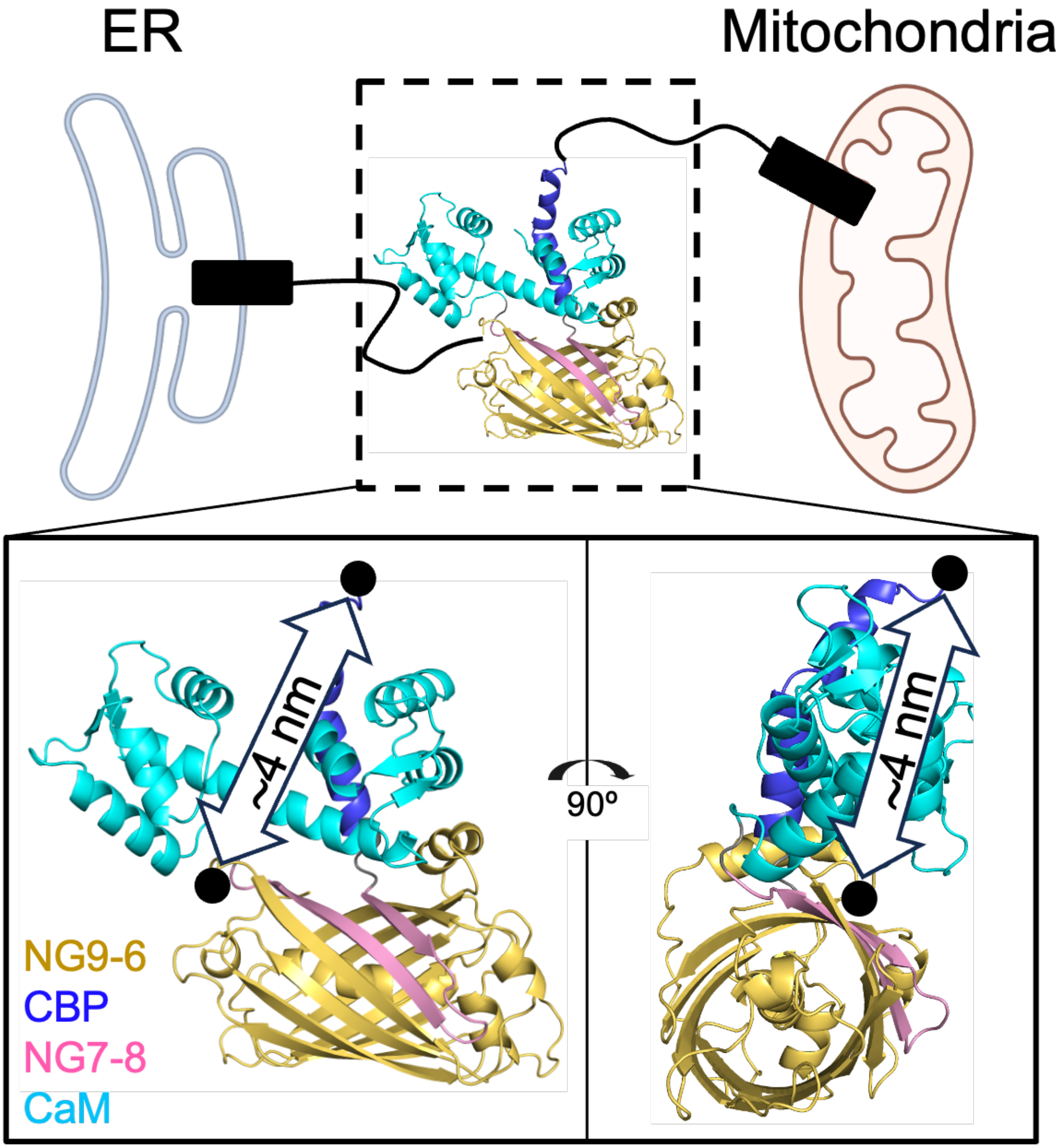

Figure S10 | AlphaFold model[1] of sN-GECO1.

SUPPLEMENTARY TABLES

Table S1 | Spectral and biophysical parameters of the split FPs and their corresponding parent FPs.

	Green		NeonGreen		Red	
	scpGFP1	EGFP	scpNG1	mNG	scpRFP1	mApple
Peak excitation wavelength (λ_{ex}) [nm]	491	488	494	504	572	569
Peak emission wavelength (λ_{em}) [nm]	510	508	516	517	597	591
Peak extinction coefficient (ε) [$mM^{-1}cm^{-1}$]	45	56	132	113	38	75
Quantum yield (Φ)	0.54	0.67	0.78	0.80	0.34	0.49
Molecular brightness	24	37.5	103	90.4	13	34.5
Apparent pK_a	7.0	6.1	5.8	5.4	7.5	6.5

Values for EGFP, mNG, and mApple are previously reported[2]. Molecular brightness is defined as the product of ε and Φ.

Table S2 | Spectral properties of previously reported FP1-10 + FP11 split FPs, and the color derivatives of scpGFP1.

	Previous reports			This work		
	Split GFP (ref. [3])	mNG3A (ref. [4])	sfCherry3C (ref. [5])	scpBFP	scpCFP	scpYFP
Peak excitation wavelength (λ_{ex}) [nm]	485	506	583	371	440	507
Peak emission wavelength (λ_{em}) [nm]	510	525	615	429	487	521
Peak extinction coefficient (ε) [$mM^{-1}cm^{-1}$]	67	180	110	39	29	5.0
Quantum yield (Φ)	0.73	0.89	0.22	0.10	0.18	0.66
Molecular brightness	49	160	24	4.0	5.2	3.3

Molecular brightness is defined as the product of ε and Φ. N.D., not determined.

Table S3 | Spectral and biophysical parameters of bipartite Ca^{2+} biosensors.

	sG-GECO1		jGCaMP7s		sN-GECO1		mNG-GECO1		sR-GECO1		R-GECO1	
Ca^{2+}	39 μM	0 μM	39 μM	0 μM	39 μM	0 μM	39 μM	0 μM	10 mM	0 μM	39 μM	0 μM
Peak excitation wavelength (λ_{ex}) [nm]	490	491	N.D.[2]	N.D.	491	492	496	N.D.	571	569	561	577
Peak emission wavelength (λ_{em}) [nm]	511	512	N.D.	N.D.	513	515	513	N.D.	598	598	589	600
Peak extinction coefficient (ε) [$mM^{-1}cm^{-1}$]	62	6.7	53	5.6	103	46	102	N.D.	71	1.5	51	15
Quantum yield (Φ)	0.88	0.79	0.65	0.58	0.63	0.20	0.69	N.D.	0.42	0.26	0.20	0.06
Molecular brightness[1]	54	5.3	35	3.2	65	9.3	70	N.D.	29	0.38	10	N.D.
Apparent pK_a	6.5	8.0	6.4	7.7	4.8	N.A.	N.D.	N.D.	7.2	8.5	6.6	8.9
$K_{d,app}$	306 nM (1.51)		68 nM		268 nM (2.35)		807 nM		3.85 μM / 1.34 mM (0.92/1.42)		167 nM	
Ca^{2+}-dependent $\Delta F/F_{min}$	10.5		39.4		17.5		35		11.2		15	

The data of sG-GECO1, sN-GECO1 and sR-GECO1 were measured in our laboratory. Data for jGCaMP7s is from ref. [6]. Data for mNG-GECO1 is from ref. [7]. Data for R-GECO1 is from ref. [8]. For sG-GECO1 and sN-GECO1, $K_{d,app}$ was calculated by fitting with a monophasic dose-response curve. For sR-GECO1, $K_{d,app}$ was calculated by fitting a biphasic dose-response curve. Molecular brightness is defined as the product of ε and Φ. Hill coefficients are displayed in parentheses. N.D., not determined.

Table S4 | One- and two-photon photophysical parameters of bipartite Ca^{2+} biosensors.

	sG-GECO1		sN-GECO1		sR-GECO1		jGCaMP7s (ref. [6])		mNG-GECO1 (ref. [7])		R-GECO1 (ref. [8])	
	Ca^{2+} free	Ca^{2+} bound	Ca^{2+} free	Ca^{2+} bound	Ca^{2+} free	Ca^{2+} bound	Ca^{2+} free	Ca^{2+} bound	Ca^{2+} free	Ca^{2+} bound	Ca^{2+} free	Ca^{2+} bound
Relative fraction of neutral chromophore (ρ_N)	0.93	0.13	N.A.	~0	0.93	0.56	0.76[a]	0.13[a]	N.A.	N.A.	0.94[a]	0.18[a]
Relative fraction of anionic chromophore (ρ_A)	0.07	0.87	N.A.	~1	0.07	0.44	0.24	0.87	N.A.	N.A.	0.06	0.82
Neutral extinction coefficient ($mM^{-1}cm^{-1}$, ε_N)	35[b]	36[b]	N.A.	N.A.	32[b]	35[b]	N.A.	N.A.	N.A.	N.A.	33	30
Anionic extinction coefficient ($mM^{-1}cm^{-1}$, ε_A)	67	69	31[b]	79[b]	75[b] 32[c]	80[b] 44[c]	5.6	53	N.A.	102	89	69
Neutral quantum yield (φ_N)	N.A.	N.A.	N.A.	N.A.	N.A.	N.A.	N.A.	N.A.	N.A.	N.A.	N.A.	N.A.
Anionic quantum yield (φ_A)	0.70	0.87	0.21	0.61	0.22	0.29	0.58	0.65	N.A.	0.69	0.15	0.21
Molecular brightness ($\rho_A \times \varepsilon_A \times \varphi_A$)	3.3[b]	52[b]	6.5[b]	48[b]	1.16[b] 0.49[c]	10[b] 5.6[c]	0.78	30	N.A.	70	0.80	11.9
Neutral 1PA peak (nm)	396	398	465	N.A.	448	448	N.A.	N.A.	N.A.	N.A.	N.A.	N.A.
Anionic 1PA peak (nm)	490	494	N.A.	488	572	573	N.A.	N.A.	N.A.	N.A.	577	563
Two-photon cross section (GM) (position/nm)	30 (900) 36 (940)	32 (932)	N.A	41 (940)	28 (1060−1130) 107 (720)	34 (1060−1130) 188 (720)	N.A.	N.A.	N.A.	N.A.	24 (1072)	31 (1056)
Two-photon brightness F_2 (GM) (position/nm)	1.8 (924)	26 (920)	3.2 (908)	27 (928)	0.43 (1060−1130) 1.65 (720)	4.3 (1060−1130) 24 (720)	N.A.	N.A.	N.A.	39[d] (970)	0.21 (1072)	5 (1056)

Mean ± s.d. N.A., not applicable.

[a]Calculated using the equation, $\log_{10}([\text{anionic}]/[\text{neutral}]) = \text{pH} - \text{p}K_a$, where pH is 7.2.

[b]Found from gradual alkaline titration.

[c]Found using the Strickler-Berg formula.

[d]Measured in ref. [7] versus fluorescein.

Table S5 | X-ray data collection and refinement statistics.

Crystal	sG-GECO1
**Data collection**	
Space group	$P4_12_12$
a, b, c (Å)	51.10, 51.10, 201.30
α, β, γ (°)	90.0, 90.0, 90.0
Resolution (Å)	36.14-1.80 (1.87-1.80)
R_{merge}	0.092
R_{meas}	0.095
Multiplicity	24.6 (24.9)
CC(1/2)	0.999 (0.626)
CC*	1 (0.877)
I/σ(I)	19.93 (1.06)
Completeness (%)	99.89 (99.13)
Wilson B-factor (Å^2)	39.68
**Refinement**	
Total Reflections	634405 (62303)
Unique Reflections	25780 (2499)
R_{work}/R_{free}	0.1838/0.2279
Number of atoms:	
Protein	1837
Ligands	22
Water	174
Average B-factor (Å^2)	44.87
Protein ADP (Å^2)	44.66
Ligands (Å^2)	33.10
Water	48.68
Ramachandran plot:	
Favored/Allowed (%)	99.10/0.90
Root-Mean-Square-Deviation:	
Bond lengths (Å)	0.008
Bond Angle (°)	1.17
PDB code	9U9D

Statistics for the highest resolution shell are shown in parentheses.

SUPPLEMENTARY METHODS

General methods and materials. Synthetic oligonucleotides were purchased from Thermo Fisher Scientific. Synthetic DNAs were purchased from Integrated DNA Technologies. Phusion High-Fidelity DNA Polymerase (Thermo Fisher Scientific) and Q5 High-Fidelity DNA Polymerase (New England Biolabs) were used for routine polymerase chain reaction (PCR) amplification. *Taq* DNA Polymerase (Invitrogen) was used for error-prone PCR. QuickChange Site-Directed Mutagenesis Kit (Agilent Technologies) was used for multiple site-directed mutagenesis. Gibson Assembly Master Mix was purchased from New England Biolabs. FastDigest Restriction Enzymes, T4 DNA Ligase, GeneJET Miniprep Kit and GeneJET Midiprep Kit were all purchased from Thermo Fisher Scientific. Products of PCR and restriction digests were purified using agarose gel electrophoresis and gel extraction by GeneJET Gel Extraction Kit (Thermo Fisher Scientific). Bacteria were transformed by electroporation (Bio-rad, #1652100). For a screening process, protein samples were expressed in *E. coli* strain DH10B (Thermo Fisher Scientific) in LB media supplemented with 100 μg mL^{-1} ampicillin and 0.02% L-arabinose and extracted using B-PER (Thermo Fisher Scientific) unless otherwise noted. Fluorescence excitation and emission spectra were recorded on Spark plate readers (Tecan). Absorption spectra were recorded on the UV-1800 Spectrometer (Shimazu). Fluorescence quantum yield (QY) was measured using the Hamamatsu Photonics absolute quantum yield spectrometer (C9920-02G). Molecular models were generated using AlphaFold[9] or AlphaFold-Multimer[10] on the ColabFold platform[11]. The FP chromophore was positioned in molecular models by superposition with the crystal structure of cpGFP (PDB ID: 3EVP)[12], mNeonGreen (PDB ID: 5LTR)[13], or mCherry (PDB ID: 2H5Q)[14].

HeLa cells were maintained in Dulbecco's modified Eagle medium (DMEM, Nacalai Tesque) containing 4.5 g L^{-1} D-glucose, supplemented with 10% (v/v) fetal bovine serum (FBS, Sigma-Aldrich) and 1% (v/v) penicillin-streptomycin (Nacalai Tesque) at 37 °C and 5% CO_2. Cells were transiently transfected with 2-3 μg of plasmid using polyethylenimine (Polysciences) in Opti-MEM (Gibco). Transfected cells were imaged 48 hours after transfection, with 10 mM HEPES-buffered Hank's balanced salt solution (HHBSS). Epifluorescence microscopy was conducted using an IX83 wide-field microscope (Olympus) using a 40× objective lens (NA = 1.30; oil), a 100× objective lens (NA = 1.45; oil) and a STR stage incubator (Tokai Hit). scpGFP1, sG-GECO1, scpNG1, sN-GECO1, Split GFP and mNG3A were imaged with a

470/20 nm excitation filter, a 490-nm dclp dichroic mirror and a 518/45 nm emission filter. scpRFP1, sR-GECO1 and sfCherry3C were imaged with a 545/20 nm excitation filter, a 565-nm dclp dichroic mirror and a 598/55 nm emission filter. Confocal microscopy was conducted using an IX81-FV1000-D confocal microscope (Olympus) using a 100× objective lens (NA = 1.35; oil). The EBFP2, scpNG1 and mApple were excited with an irradiation of 405 nm, 488 nm and 559 nm laser lights, respectively. Fluorescence images were analyzed with ImageJ software (National Institutes of Health) and custom python scripts.

Two-photon characterization of sGECOs. Two-photon excitation (2PE) spectra were measured as described[15]. In the spectral shape measurements, a combination of 770SP, 680SP and 633SP filters was used to block the laser scattering. The cross-section $\sigma_{2,R}$ of the red forms were measured at a particular wavelength λ_{2P}, falling in the range of only red form absorption, as previously described[16]. Rhodamine 6G (Rh6G) in methanol or fluorescein in water at pH 12 were used as a reference standard. To obtain the two-photon excitation spectra in units of molecular brightness, the unscaled 2PE spectra were normalized to the product $F_{2,R} = \varphi_R \times \sigma_{2,R} \times \rho_R$ measured at a wavelength λ_{2P}. To measure the signal-to-background ratio upon saturating with Ca^{2+}, the fluorescence signals (either one-photon or two-photon) were measured in the Ca^{2+}-free and Ca^{2+}-saturated samples with the same concentration of protein and under the same conditions.

X-ray crystallography. sG-GECO1 recombinant protein was purified with size exclusion chromatography and concentrated to 28 mg mL^{-1} and used for crystallization trials. Initial crystallization was set up with 384 well plates via sitting drop vapor diffusion against commercially available kits at 20 °C. The sG-GECO1 protein crystals were grown in the buffer of 0.2 M sodium malonate dibasic monohydrate, 0.1 M bis-tris propane (pH 8.5), 20% w/v PEG 3350. The crystals were cryo protected with a reservoir supplemented with 20% glycerol in liquid nitrogen for X-ray diffraction. X-ray diffraction data were collected at Shanghai Synchrotron Radiation Facility (SSRF) Beamline BL18U1. The X-ray diffraction data were scaled using XDS[17]. Data collection statistics are summarized in **Table S5**. The sG-GECO1 belonged to the tetragonal space group $P4_12_12$ with one sG-GECO1 in the asymmetric unit. Molecular replacement was performed with Phaser using GFP protein as search model[18,19]. The final datasets were processed to 1.8 Å with a unit cell parameter of a = 51.10 Å, b = 51.10 Å and c = 201.30 Å. Structure model building and refinement were carried out in Coot and

Phenix program[20,21]. The whole GFP molecule was found with only the linker region and partial residues were traced from the Ca^{2+} sensory domain. The final model demonstrated a R_{work}/R_{free} value of 0.1838/0.2279.

Construction of mammalian expression plasmids. pcDNA-MBP-spFPs (**Figure 3d,e,g,h,j,k**): To express our new scpFPs in living mammalian cells, we transferred the genes of scpGFP1, scpNG1 and scpRFP1 into the expression vector pcDNA3.1(+). The genes encoding MBP-FP7-8 was amplified by PCR with P2A self-cleaving peptide sequence (ATNFSLLKQAGDVEENPGP)[22], and digested with XhoI/EcoRI. The gene encoding FP9-6 was amplified by PCR and cut with EcoRI/HindIII. Finally, both genes were enzymatically ligated into pcDNA3.1(+) plasmid.

pcDNA-MBP-SplitGFP/mNG3A/sfCherry3C (**Figure 3e,h,k**): Using synthesised Split GFP[3], mNG3A[4], and sfCherry3C[5], we constructed co-expression plasmids for themas described above.

pcDNA-scpFP each fragment (**Figure S6a**–**f**): To express each fragment of scpFPs from separate plasmids, we constructed pcDNA plasmids having either MBP-FP7-8 or FP9-6 by PCR, enzymatic digestion with XhoI/HindIII and ligation into pcDNA plasmids.

Organelle targeting plasmids (**Figure S6g**–**v**): To express our new scpFPs at different organelles, we fused a small fragment of scpFPs to the different target proteins. We used genes encoding keratin (Addgene #54134), laminB1 (Addgene #55425), actin (Addgene #56400), histone H2B (Addgene #56651), vimentin (Addgene #56654), TOM20 (a gift from Prof. Takeaki Ozawa in The University of Tokyo) and calnexin (synthesized, Uniplot #27824), as the templates. The gene encoding each FP7-8 was fused to N-terminus of laminB1 and actin, and C-terminus of keratin, histone H2B, vimentin, TOM20 and calnexin, by Gibson assembly.

pcDNA-sGECOs: To test our new bipartite Ca^{2+} biosensors in living mammalian cells, we transferred the genes of s-GECOs into expression vector pcDNA3.1(+). The first fragment containing CBP-FP7-8-CaM was amplified by PCR with P2A sequence and digested either with XhoI/BglII or XhoI/KpnI. The second fragment containing FP9-6 was amplified and digested either with BglII/HindIII or KpnI/HindIII. Both fragments were enzymatically ligated into pcDNA plasmids.

Imaging of cytosolic sGECOs in HeLa cells. For imaging of the histamine-induced cytosolic Ca^{2+} oscillation, fluorescence images were acquired every 5 seconds for a duration of 20

minutes. One minute after the start of the experiments, 5 μM (final concentration) of histamine in HHBSS(+) was added. The oscillations were imaged for 10 minutes. After 15 minutes from the start of the experiment, 1 mM of EGTA and 1.5 μM (final concentration) of ionomycin were added to chelate Ca^{2+}, followed by adding 2 mM of $CaCl_2$ and 0.75 μM (final concentration) of ionomycin to turn sGECOs on again. For imaging of the thapsigargin-induced Ca^{2+} release from ER, fluorescence images were acquired every 5 seconds for a duration of 20 minutes. Five minutes after the start of the experiments, 5 μM (final concentration) of thapsigargin in HHBSS(+) was added.

Constructs for targeting of ER–mitochondria contact sites. To generate the SPLICa:ER-MT constructs, we firstly amplified the NG9-6 fragment and we inserted it into the plasmid pcDNA3-GFP_{1-10}-ER[23,24], using EcoRI and XhoI. The construct that we obtained is pcDNA3-NG9-6-ER (1×), with a connecting region of 18 aa between the NG9-6 and the ER targeting sequence. To obtain the pcDNA3-NG9-6-ER 2× and 3×, we amplified the NG9-6 fragment with a reverse primer containing either one copy or two copies of the connecting region, and we performed the same cloning strategy as for pcDNA3-NG9-6-ER (1×). In the resulting pcDNA3-NG9-6-ER 2× and 3×, the connecting region between the NG9-6 and the ER targeting sequence is 34 and 48 aa long, respectively. Secondly, we replaced the whole sequence of TOM20 in the plasmid pcDNA3.1-TOM20-CBP-FP7-8-CaM with its minimal mitochondrial targeting sequence (MTS, first 33 aa). We amplified the MTS and inserted it using NheI and EcoRI. The generation of the single bicistronic vector containing the NG9-6-ER 3× and the TOM20 MTS-CBP-FP7-8-CaM was carried out by Genescript.

Cell culture for imaging of ER–mitochondria contact sites. HeLa cells (ATCC) were grown at 37 °C in a 5% CO_2 atmosphere in DMEM high glucose (Gibco; #41966-029), supplemented with 10% FBS (Gibco; #10270-106), 100 U mL^{-1} penicillin and 100 mg mL^{-1} streptomycin (Penicillin–Streptomycin solution 100×) (EuroClone; #ECB3001D).

HeLa cells were transfected by standard Ca^{2+} phosphate protocol. Briefly, for one 13 mm coverslip, 5 μL of 2.5 M $CaCl_2$ (Sigma-Aldrich; #C-5080) was added to 1 μg of total SPLICa:ER-MT probe DNA dissolved in Milli-Q H_2O to reach a final volume of 50 μL. $CaCl_2$-DNA solution was added drop by drop to 50 μL HEPES Buffered Solution 2× (HBS 2×: 280 mM NaCl, 50 mM HEPES, 1.5 mM $Na_2HPO_4{\cdot}7H_2O$ (Sigma-Aldrich; #S9390), pH 7.12) and incubated 30 min at room temperature (RT). Before transfection, the growth medium was

replaced with fresh medium. Eight-hours after transfection cells were washed three times with Dulbecco's Phosphate Buffered Saline (D-PBS) (EuroClone; #ECB4004L) to remove excess of Ca^{2+} phosphate precipitates and fresh medium was replaced, incubating cells for additional 24 hours.

Immunocytochemistry of ER–mitochondria contact sites. Twenty-four hours post transfection cells, plated on 13-mm glass coverslips, were fixed for 20 min in a 3.7% (vol/vol) formaldehyde solution (Sigma-Aldrich; #F8775). Cells were then washed three times with D-PBS (Euroclone). Cell permeabilization was performed by 10 min incubation in 0.3% Triton X-100 Bio-Chemica (PanReac AppliChem; #A1388) in D-PBS, followed by three times washes in 1% gelatin/D-PBS (Type B from bovine skin) (Sigma-Aldrich; #G9382) for 15 min at RT. The coverslips were then incubated for 90 min at RT with the specific primary antibody diluted in D-PBS (1:50 anti-KDEL, abcam; #2898). Three washing with 1% gelatine/D-PBS were performed to remove the excess of primary antibody. Staining was revealed by the incubation with a dilution 1:100 in D-PBS of specific Alexa Fluor secondary antibodies (Thermo Fisher Scientific: Goat anti-Rabbit IgG Alexa Fluor 647) for 45 min at RT. After three additional washing with 1% gelatine/D-PBS, coverslips were mounted using Mowiol 40-88 (Sigma-Aldrich; #81386). Mitochondria of transfected HeLa were stained by MitoTracker™ Red CMXRos (Invitrogen; #M7512) in accordance with the manufacturer's instructions. Briefly, before fixing, cells were washed in HBSS (GIBCO) and incubated with 150 nM MitoTracker Red CMXRos (Invitrogen; #9082), for 15 minutes at 37 °C in a 5% CO_2 atmosphere.

REFERENCES

(1) Abramson, J.; Adler, J.; Dunger, J.; Evans, R.; Green, T.; Pritzel, A.; Ronneberger, O.; Willmore, L.; Ballard, A. J.; Bambrick, J.; Bodenstein, S. W.; Evans, D. A.; Hung, C.-C.; O'Neill, M.; Reiman, D.; Tunyasuvunakool, K.; Wu, Z.; Žemgulytė, A.; Arvaniti, E.; Beattie, C.; Bertolli, O.; Bridgland, A.; Cherepanov, A.; Congreve, M.; Cowen-Rivers, A. I.; Cowie, A.; Figurnov, M.; Fuchs, F. B.; Gladman, H.; Jain, R.; Khan, Y. A.; Low, C. M. R.; Perlin, K.; Potapenko, A.; Savy, P.; Singh, S.; Stecula, A.; Thillaisundaram, A.; Tong, C.; Yakneen, S.; Zhong, E. D.; Zielinski, M.; Žídek, A.; Bapst, V.; Kohli, P.; Jaderberg, M.; Hassabis, D.; Jumper, J. M. Accurate Structure Prediction of Biomolecular Interactions with AlphaFold 3. *Nature* **2024**, *630*, 493–500.

(2) Cranfill, P. J.; Sell, B. R.; Baird, M. A.; Allen, J. R.; Lavagnino, Z.; de Gruiter, H. M.; Kremers, G.-J. J.; Davidson, M. W.; Ustione, A.; Piston, D. W. Quantitative Assessment of Fluorescent Proteins. *Nat. Methods* **2016**, *13* (7), 557–562.

(3) Cabantous, S.; Terwilliger, T. C.; Waldo, G. S. Protein Tagging and Detection with Engineered Self-Assembling Fragments of Green Fluorescent Protein. *Nat. Biotechnol.* **2005**, *23* (1), 102–107.

(4) Zhou, S.; Feng, S.; Brown, D.; Huang, B. Improved Yellow-Green Split Fluorescent Proteins for Protein Labeling and Signal Amplification. *PLoS One* **2020**, *15* (11), e0242592.

(5) Feng, S.; Varshney, A.; Coto Villa, D.; Modavi, C.; Kohler, J.; Farah, F.; Zhou, S.; Ali, N.; Müller, J. D.; Van Hoven, M. K.; Huang, B. Bright Split Red Fluorescent Proteins for the Visualization of Endogenous Proteins and Synapses. *Commun. Biol.* **2019**, *2*, 344.

(6) Dana, H.; Sun, Y.; Mohar, B.; Hulse, B. K.; Kerlin, A. M.; Hasseman, J. P.; Tsegaye, G.; Tsang, A.; Wong, A.; Patel, R.; Macklin, J. J.; Chen, Y.; Konnerth, A.; Jayaraman, V.; Looger, L. L.; Schreiter, E. R.; Svoboda, K.; Kim, D. S. High-Performance Calcium Sensors for Imaging Activity in Neuronal Populations and Microcompartments. *Nat. Methods* **2019**, *16* (7), 649–657.

(7) Zarowny, L.; Aggarwal, A.; Rutten, V. M. S.; Kolb, I.; GENIE Project; Patel, R.; Huang, H.-Y.; Chang, Y.-F.; Phan, T.; Kanyo, R.; Ahrens, M. B.; Allison, W. T.; Podgorski, K.; Campbell, R. E. Bright and High-Performance Genetically Encoded Ca2+ Indicator Based on MNeonGreen Fluorescent Protein. *ACS Sens.* **2020**, *5* (7), 1959–1968.

(8) Zhao, Y.; Araki, S.; Wu, J.; Teramoto, T.; Chang, Y.-F.; Nakano, M.; Abdelfattah, A. S.; Fujiwara, M.; Ishihara, T.; Nagai, T.; Campbell, R. E. An Expanded Palette of Genetically Encoded Ca^{2+} Indicators. *Science* **2011**, *333* (6051), 1888–1891.

(9) Jumper, J.; Evans, R.; Pritzel, A.; Green, T.; Figurnov, M.; Ronneberger, O.; Tunyasuvunakool, K.; Bates, R.; Žídek, A.; Potapenko, A.; Bridgland, A.; Meyer, C.; Kohl, S. A. A.; Ballard, A. J.; Cowie, A.; Romera-Paredes, B.; Nikolov, S.; Jain, R.; Adler, J.; Back, T.; Petersen, S.; Reiman, D.; Clancy, E.; Zielinski, M.; Steinegger, M.; Pacholska,

M.; Berghammer, T.; Bodenstein, S.; Silver, D.; Vinyals, O.; Senior, A. W.; Kavukcuoglu, K.; Kohli, P.; Hassabis, D. Highly Accurate Protein Structure Prediction with AlphaFold. *Nature* **2021**, *596* (7873), 583–589.

(10) Evans, R.; O'Neill, M.; Pritzel, A.; Antropova, N.; Senior, A.; Green, T.; Žídek, A.; Bates, R.; Blackwell, S.; Yim, J.; Ronneberger, O.; Bodenstein, S.; Zielinski, M.; Bridgland, A.; Potapenko, A.; Cowie, A.; Tunyasuvunakool, K.; Jain, R.; Clancy, E.; Kohli, P.; Jumper, J.; Hassabis, D. Protein Complex Prediction with AlphaFold-Multimer. *bioRxiv*, **2021**. https://doi.org/10.1101/2021.10.04.463034.

(11) Mirdita, M.; Schütze, K.; Moriwaki, Y.; Heo, L.; Ovchinnikov, S.; Steinegger, M. ColabFold: Making Protein Folding Accessible to All. *Nat. Methods* **2022**, *19* (6), 679–682.

(12) Wang, Q.; Shui, B.; Kotlikoff, M. I.; Sondermann, H. Structural Basis for Calcium Sensing by GCaMP2. *Structure* **2008**, *16* (12), 1817–1827.

(13) Clavel, D.; Gotthard, G.; von Stetten, D.; De Sanctis, D.; Pasquier, H.; Lambert, G. G.; Shaner, N. C.; Royant, A. Structural Analysis of the Bright Monomeric Yellow-Green Fluorescent Protein MNeonGreen Obtained by Directed Evolution. *Acta Crystallogr. D Struct. Biol.* **2016**, *72* (Pt 12), 1298–1307.

(14) Shu, X.; Shaner, N. C.; Yarbrough, C. A.; Tsien, R. Y.; Remington, S. J. Novel Chromophores and Buried Charges Control Color in MFruits. *Biochemistry* **2006**, *45* (32), 9639–9647.

(15) Drobizhev, M.; Molina, R. S.; Hughes, T. E. Characterizing the Two-Photon Absorption Properties of Fluorescent Molecules in the 680-1300 Nm Spectral Range. *Bio Protoc.* **2020**, *10* (2), e3498.

(16) Dalangin, R.; Jia, B. Z.; Qi, Y.; Aggarwal, A.; Sakoi, K.; Drobizhev, M.; Molina, R. S.; Patel, R.; Abdelfattah, A. S.; Zheng, J.; Reep, D.; Hasseman, J. P.; GENIE Project Team; Zhao, Y.; Wu, J.; Podgorski, K.; Tebo, A. G.; Schreiter, E. R.; Hughes, T. E.; Terai, T.; Paquet, M.-E.; Megason, S. G.; Cohen, A. E.; Shen, Y.; Campbell, R. E. Far-Red Fluorescent Genetically Encoded Calcium Ion Indicators. *Nat. Commun.* **2025**, *16* (1), 3318.

(17) Kabsch, W. XDS. *Acta Crystallogr. D Biol. Crystallogr.* **2010**, *66* (Pt 2), 125–132.

(18) McCoy, A. J. Solving Structures of Protein Complexes by Molecular Replacement with Phaser. *Acta Crystallogr. D Biol. Crystallogr.* **2007**, *63* (Pt 1), 32–41.

(19) Wu, S.-Y.; Wen, Y.; Serre, N. B. C.; Laursen, C. C. H.; Dietz, A. G.; Taylor, B. R.; Drobizhev, M.; Molina, R. S.; Aggarwal, A.; Rancic, V.; Becker, M.; Ballanyi, K.; Podgorski, K.; Hirase, H.; Nedergaard, M.; Fendrych, M.; Lemieux, M. J.; Eberl, D. F.; Kay, A. R.; Campbell, R. E.; Shen, Y. A Sensitive and Specific Genetically-Encoded Potassium Ion Biosensor for in Vivo Applications across the Tree of Life. *PLoS Biol.* **2022**, *20* (9), e3001772.

(20) Emsley, P.; Cowtan, K. Coot: Model-Building Tools for Molecular Graphics. *Acta Crystallogr. D Biol. Crystallogr.* **2004**, *60* (Pt 12 Pt 1), 2126–2132.

(21) Adams, P. D.; Afonine, P. V.; Bunkóczi, G.; Chen, V. B.; Davis, I. W.; Echols, N.; Headd, J. J.; Hung, L.-W.; Kapral, G. J.; Grosse-Kunstleve, R. W.; McCoy, A. J.; Moriarty, N. W.; Oeffner, R.; Read, R. J.; Richardson, D. C.; Richardson, J. S.; Terwilligere, T. C.; Zwarta, P. H.. Phenix - a Comprehensive Python-Based System for Macromolecular Structure Solution. *Acta Crystallogr. D Struct. Biol.* **2010**, *66* (2), 213–221.

(22) Liu, Z.; Chen, O.; Wall, J. B. J.; Zheng, M.; Zhou, Y.; Wang, L.; Vaseghi, H. R.; Qian, L.; Liu, J. Systematic Comparison of 2A Peptides for Cloning Multi-Genes in a Polycistronic Vector. *Sci. Rep.* **2017**, *7* (1), 2193.

(23) Cieri, D.; Vicario, M.; Giacomello, M.; Vallese, F.; Filadi, R.; Wagner, T.; Pozzan, T.; Pizzo, P.; Scorrano, L.; Brini, M.; Calì, T. SPLICS: A Split Green Fluorescent Protein-Based Contact Site Sensor for Narrow and Wide Heterotypic Organelle Juxtaposition. *Cell Death Differ.* **2018**, *25* (6), 1131–1145.

(24) Vallese, F.; Catoni, C.; Cieri, D.; Barazzuol, L.; Ramirez, O.; Calore, V.; Bonora, M.; Giamogante, F.; Pinton, P.; Brini, M.; Calì, T. An Expanded Palette of Improved SPLICS Reporters Detects Multiple Organelle Contacts in Vitro and in Vivo. *Nat. Commun.* **2020**, *11* (1), 6069.
